# Histaminylation alters collagen matrix mechanics and attenuates cardiac fibrosis post-myocardial infarction via mechanotransduction signaling axis

**DOI:** 10.1038/s41392-026-02721-5

**Published:** 2026-06-11

**Authors:** Jianfu Zhu, Dili Sun, Gaofeng Zeng, Kehan Wu, Xiyang Yang, Xiaowei Zhu, Diyaerjiang Aierken, Suling Ding, Xiangfei Wang, Junbo Ge, Xiangdong Yang

**Affiliations:** 1 Shanghai Institute of Cardiovascular Diseases, Zhongshan Hospital, Fudan University, Shanghai, China; 2 Department of Cardiology, Zhongshan Hospital, Fudan University, Shanghai, China; 3 NHC Key Laboratory of Ischemic Heart Diseases, State Key Laboratory of Cardiovascular Diseases, Zhongshan Hospital, Fudan University, Shanghai, China; 4 Department of Cardiology, the Second Affiliated Hospital, Hengyang Medical School, Key Laboratory of Heart Failure Prevention & Treatment of Hengyang, Clinical Medicine Research Center of Arteriosclerotic Disease of Hunan Province, University of South China, Hengyang, China; 5 Department of Critical Care Medicine, Zhongshan Hospital, Fudan University, Shanghai, China

**Keywords:** Cell biology, Biomaterials

## Abstract

Histaminylation is a newly discovered monoaminylation catalyzed by transglutaminase 2 (TGM2), and its role in myocardial fibrosis has not yet been elucidated. Here, we identified histaminylation in cardiac type I collagen isolated from mice 7 days after acute myocardial infarction (AMI) by mass spectrometry. Using histamine-deficient *Hdc*^−/−^ mice, we demonstrated an increase in TGM2-mediated γ-glutamyl-ε-lysine crosslinks and a higher myocardial Young’s modulus in the scar region of *Hdc*^−/−^ mice than in wild-type controls, indicating that histaminylation might restrain the role of TGM2 in cardiac fibrogenesis after AMI. Furthermore, we reconstituted an in vitro crosslinking system, yielding unmodified collagen and histaminylated collagen. The results of mechanical indentation and electron microscopy demonstrated that histaminylation could reduce crosslink formation, lower matrix stiffness, and alter viscoelasticity. Subsequently, primary murine cardiac fibroblasts were cultured under cyclic stretch with TGF-β on different substrates, and the results revealed that histaminylated collagen strongly attenuated TGF-β-induced fibroblast-to-myofibroblast transition and reduced focal adhesion assembly. Mechanically, integrated single-cell and bulk RNA-seq analyses indicated that the PIEZO1/ITGB1 mechanotransduction axis was upregulated and mediated downstream signal transduction in the infarcted hearts of *Hdc*^−/−^ mice. Finally, local delivery of a histamine-releasing hydrogel to *Hdc*^−/−^ mice after AMI could significantly reinstate collagen histaminylation and reduce crosslink abundance and cardiac dysfunction. Collectively, these data reveal that collagen histaminylation limits TGM2-dependent crosslinking, softens the extracellular matrix, and inhibits the PIEZO1/ITGB1 axis, thereby mitigating myocardial fibrosis after AMI. This study also highlights a novel role of histamine in maintaining stress environment mechanical stability.

## Introduction

Fibrosis constitutes a key pathological hallmark across a broad spectrum of cardiac disorders, encompassing both hereditary cardiomyopathies and ischemic heart disease.^1^ A unifying feature of most fibrotic hearts is the mechanical stiffening that arises from excessive deposition of extracellular matrix (ECM).^2^ Although this increased stiffness arises directly from fibrotic changes, it also acts as a powerful activation cue for cardiac fibroblasts (CFs), inducing their transition into myofibroblasts as vital effectors of fibrotic remodeling.^3^ Type I collagen, as the principal constituent of the cardiac ECM, exerts a critical influence on ECM stiffness and other mechanical properties, such as viscoelasticity, through its crosslinking.^4^ Currently, three well-defined factors have been identified to mediate collagen crosslinking: lysyl oxidase (LOX), transglutaminase 2 (TGM2), and advanced glycation end-products (AGEs).^5^ Among them, TGM2 enzymatically catalyzes the formation of γ-glutamyl-ε-lysine (γ-Glu-ε-Lys) isopeptide crosslinks between glutamine (Gln) residues and lysine (Lys) residues, thereby enhancing the stability, irreversibility, and resistance to remodeling of collagen scars.^6^ Currently, studies on TGM2 in acute myocardial infarction (AMI) are limited and poorly understood.

Beyond its classical role in collagen crosslinking, TGM2 also mediates a novel type of post-translational modification (PTM) known as monoaminylation.^7^ This modification involves a transamidation reaction through which monoamine compounds, including serotonin, dopamine, histamine, and norepinephrine, are covalently attached to the Gln residues of protein substrates.^8^ Histaminylation, a specific form of monoaminylation, refers to the covalent attachment of histamine to the Gln residues of target proteins.^9^ Histamine, an important monoamine bioactive molecule, is produced through the decarboxylation of L-histidine by histidine decarboxylase (HDC).^10^ Previous studies have demonstrated that histamine plays multiple roles in various pathophysiological processes, including gastric acid secretion, allergy, inflammatory reactions, and immune cell differentiation.^11,12^ Using *Hdc*-GFP transgenic mice and immunofluorescence techniques, studies revealed that *Hdc* is highly expressed in *Cd11b*^+^ myeloid cells residing in the bone marrow and spleen, rather than in cardiomyocytes, endothelial cells, and CFs.^13,14^ Recent studies reported that histamine deficiency or the administration of histamine receptor (HR) 1 antagonists could significantly aggravate cardiomyocyte apoptosis and cardiac fibrosis in response to AMI and doxorubicin treatment, respectively.^15,16^ However, to the best of our knowledge, the role and underlying mechanisms of histaminylation in cardiac fibrosis following AMI remain largely unexplored.

A large number of *Hdc*-expressing *Cd11b*^+^*Ly6g*^+^ neutrophils and *Cd11b*^+^*Ly6c*^+^ monocytes/macrophages were recruited to the injured hearts and locally synthesized and released histamine.^17^ This leads us to speculate whether histamine plays other important roles in the extracellular microenvironment in addition to binding to HRs on the cell membrane. Notably, both collagen crosslinking and histaminylation occur at the same site, Gln residues on collagen fibrils, suggesting that histaminylation may interfere with or modulate collagen crosslinking.^18^ This regulatory effect may further influence cellular behavior by altering the stiffness and viscoelasticity of the ECM, thereby affecting the fibrotic process. Investigating how this novel PTM modulates the mechanical properties of the ECM could provide casual insights into how histamine, through nonreceptor-mediated mechanisms, contributes to cardiac fibrosis following ischemic injury.

In this study, we first identified the expression of histaminylation in cardiac type I collagen isolated from mice 7 days after AMI by liquid chromatography-tandem mass spectrometry (LC-MS/MS). Using histamine-deficient *Hdc* knockout (*Hdc*^−/−^) mice, we demonstrated an increase in TGM2-mediated γ-Glu-ε-Lys crosslinks and a higher myocardial Young’s modulus in the injured hearts of *Hdc*^−/−^ mice compared to wild-type (WT) controls, indicating that the abnormal ablation of histaminylation might promote the role of TGM2 in cardiac fibrogenesis after AMI. To further investigate the mechanical alterations of the collagen matrix and underlying mechanisms, we established an in vitro collagen matrix crosslinked by TGM2 and modified with histamine. LC-MS/MS data confirmed the presence of prominent histaminylation upon histamine addition. Notably, histaminylated collagen exhibited a significant reduction in Young’s modulus, altered viscoelasticity, and enhanced degradability, resulting in the significant suppression of fibroblast-to-myofibroblast transition (FMT). This suppression was associated with reduced PIEZO1-mediated calcium influx, decreased focal adhesion (FA) formation, and downregulated *Itgb1* expression. Finally, we demonstrated that local delivery of a histamine-releasing hydrogel to *Hdc*^−/−^ mice after AMI could reinstate collagen histaminylation and significantly reduce crosslink abundance and cardiac dysfunction.

## Results

### Histaminylation is identified in type I collagen in the post-AMI mouse heart

**Fig. 1:**
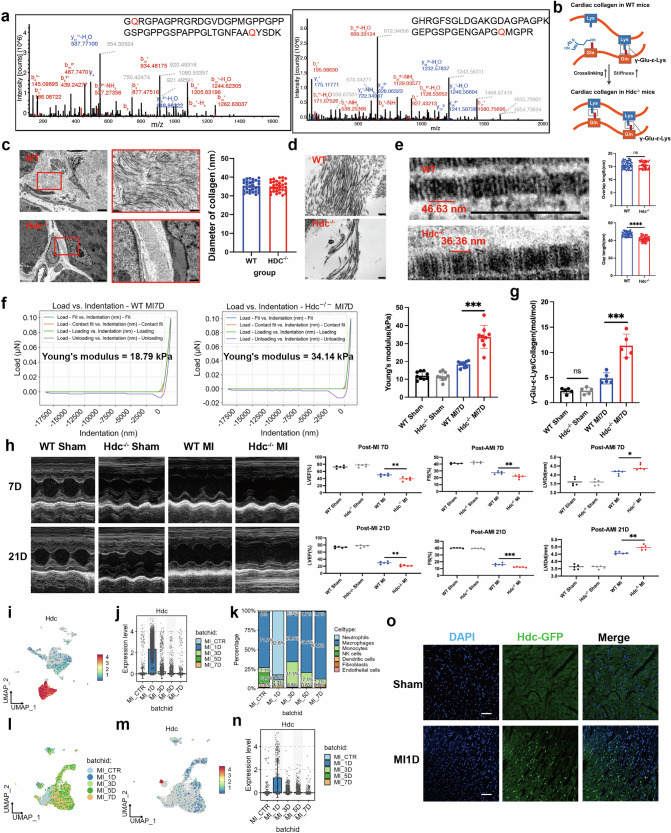
The detection and functional analysis of histaminylation in type I collagen in cardiac tissue after AMI. **a** LC-MS/MS analysis of histaminylated type I collagen showed multiple histaminylation modifications on Gln(Q) residues. **b** Schematic model illustrating that histaminylation may inhibit TGM2-mediated collagen crosslinking by occupying Gln residues. **c** TEM images of cardiac collagen fibers from WT and *Hdc*^−/−^ mice on day 7 post-AMI (scale bars: left, 2 μm; right, 500 nm), and quantification analysis showing no significant difference in the diameter of individual collagen fibrils between WT and *Hdc*^−/−^ mice (randomly selected fibrils, *n* ≥ 30). **d** Both WT and *Hdc*^−/−^ mice exhibited well-defined D-periods in collagen fibrils (scale bar: 200 nm). **e** The gap region of the D-periods was significantly shortened in collagen fibrils from *Hdc*^−/−^ mice compared to WT mice, while the overlap region remained unchanged (randomly selected, *n* = 50; scale bar: 200 nm). **f** Representative and quantitative analysis of indentation curves showing significantly increased Young’s modulus in *Hdc*^−/−^ infarcted myocardium scars (34.14 kPa) compared to WT controls (18.79 kPa) (*n* = 9). **g** AQMC analysis revealed a significant increase in the TGM2 crosslinking product γ-Glu-ε-Lys in the cardiac tissue of *Hdc*^−/−^ mice relative to WT controls (*n* = 5). **h** Representative image and quantitative analysis of echocardiography on at day 7 and day 21 post-AMI (*n* = 5). **i** Dimplot of Hdc expression levels across all cell types, showing predominant expression in neutrophils and a subset of macrophages. **j** Barplot of *Hdc* expression in the total cell population, indicating peak expression on day 1 post-AMI and elevated levels on day 3. **k** The proportion of *Hdc*^high^ neutrophils peaks on day 1 post-AMI. **l** Re-clustering dimplot of macrophages from the dataset, grouped by time point post-AMI. **m** Dimplot identification of a macrophage subcluster with a high level of *Hdc* expression. **n** Barplot of *Hdc* expression in macrophages, showing a peak on day 1 and elevated levels on day 3 post-AMI. **o** Cardiac tissue section from *Hdc*-GFP mice on day 1 post-AMI showing GFP^+^ cells migrating into the injured region. For all experiments, error bars represent the mean ± SD. *P < 0.05, **P < 0.01, ***P < 0.001, ****P < 0.0001

There have been no previous reports of histaminylation of collagen in cardiac tissue following AMI. To identify the presence of histaminylation in the ECM, we employed a previously reported pepsin-digestion method^19^ to extract collagen from the hearts of WT and *Hdc*^−/−^ mice 7 days after AMI (Supplementary Fig. 1a, b). The extracted collagen was subsequently purified by sodium dodecyl sulfate-polyacrylamide gel electrophoresis (SDS-PAGE) and subjected to LC-MS/MS analysis specifically targeting histaminylation (Supplementary Fig. 1c). The results of LC-MS/MS analysis showed multiple histaminylation modifications on Gln residues in cardiac type I collagen of WT mice (Fig. 1a, and Supplementary Dataset 1, 2). We hypothesize that histaminylation occupies specific Gln residues and thereby prevents collagen crosslinking (Fig. 1b). To validate this hypothesis, transmission electron microscope (TEM) was used to investigate the effect of histaminylation on the structure of collagen fibrils in the infarcted cardiac tissue of WT and *Hdc*^−/−^ mice 7 days post AMI. The TEM results showed that collagen fibers in *Hdc*^−/−^ mice appeared more densely packed and orderly arranged than those in WT mice (Fig. 1c). However, no significant difference in fibrillar collagen diameter was observed between the two groups (Fig. 1c). Subsequently, we investigated the collagen-specific D-period structure associated with collagen fibril crosslinking. Fibrillar collagen from *Hdc*^−/−^ mice exhibited the characteristic alternating light and dark D-period pattern typical of type I collagen, similar to that observed in WT mice (Fig. 1d). However, quantitative analysis revealed a significant reduction in the average length of the gap region of the D-period in *Hdc*^−/−^ mice, while the average length of the overlap region remained unchanged (Fig. 1e). The results of the mechanical property analysis of cardiac tissue further demonstrated that the infarct region distal to the ligation site in *Hdc*^−/−^ mice exhibited a significantly higher Young’s modulus than WT mice on day 7 post AMI, indicating that increased tissue stiffness likely resulted from excessive collagen crosslinking (Fig. 1f). We then employed a previously reported and validated absolute quantification of matrix-specific crosslinking (AQMC) method^20^ to assess the extent of TGM2-mediated crosslinking in collagen fibrils. This method effectively eliminates the interference caused by the absolute collagen content, enabling accurate quantitative analysis of the degree of collagen crosslinking. The AQMC data revealed a significant increase in the TGM2-catalyzed crosslinking product γ-Glu-ε-Lys in the infarcted cardiac tissue of *Hdc*^−/−^ mice compared to WT controls 7 days post AMI (Fig. 1g). In addition, the results of echocardiographic assessment, including left ventricular ejection fraction (LVEF), fractional shortening (FS), left ventricular end-diastolic internal dimension (LVIDd), and Masson’s trichrome staining, confirmed that *Hdc*^−/−^ mice exhibited significantly exacerbated cardiac fibrosis and impaired cardiac function compared with WT mice at 21 days post AMI (Fig. 1h, and Supplementary Fig. 1d). Collagen extractability in *Hdc*^−/−^ mice was significantly lower than that in WT mice when extracted from the same weight of cardiac tissue (Supplementary Fig. 2). Collectively, these data suggest the critical roles of histamine and TGM2-mediated histaminylation in cardiac fibrogenesis after AMI.

To identify the cellular source of histamine post-AMI, we analyzed a *Cd45*^+^ cell-enriched single-cell RNA-seq (scRNA-seq) dataset.^21^ *Hdc* expression was mainly detected in neutrophils and a subset of macrophages, peaking on day 1 post AMI, declining by day 3, and absent in controls (Fig. 1i–n, and Supplementary Fig. 3a). Neutrophils dominated early, while macrophages prevailed from day 3–7 (Fig. 1k). Reclustering revealed a rare MI-specific *Hdc*^+^*Cxcr2*^+^ macrophage subset, suggesting a histamine-producing phenotype (Fig. 1l–n, and Supplementary Figs. 3b and 4). The immunofluorescence and flow cytometry results confirmed that *Hdc*^+^ cells were significantly increased in the bone marrow, blood, and infarcted hearts of *Hdc*-GFP mice on day 1 (Fig. 1o, and Supplementary Fig. 3c). These findings indicate that histamine-producing myeloid cells are enriched in the early 1–7 days post AMI phase.

Measurement of histamine, dopamine, and serotonin on day 3 post AMI showed a selective histamine increase in the serum and myocardium of WT mice, which was absent in *Hdc*^−/−^ mice, without changes in dopamine or serotonin (Supplementary Fig. 5a–c). Bulk RNA-seq revealed no genotype-related differences in *Tgm2* or *Lox* expression, both following similar temporal patterns (Supplementary Fig. 5d–f). Metabolomics showed comparable glucose levels between genotypes, excluding excessive AGE-mediated crosslinking (Supplementary Fig. 5g). Thus, histamine deficiency, rather than other monoamines or known enzymatic crosslinkers, likely underlies the aggravated fibrosis and abnormal collagen structure in *Hdc*^−/−^ mice after AMI.

### Histaminylation is present in an in vitro-constructed collagen matrix and compromises its mechanical properties

**Fig. 2:**
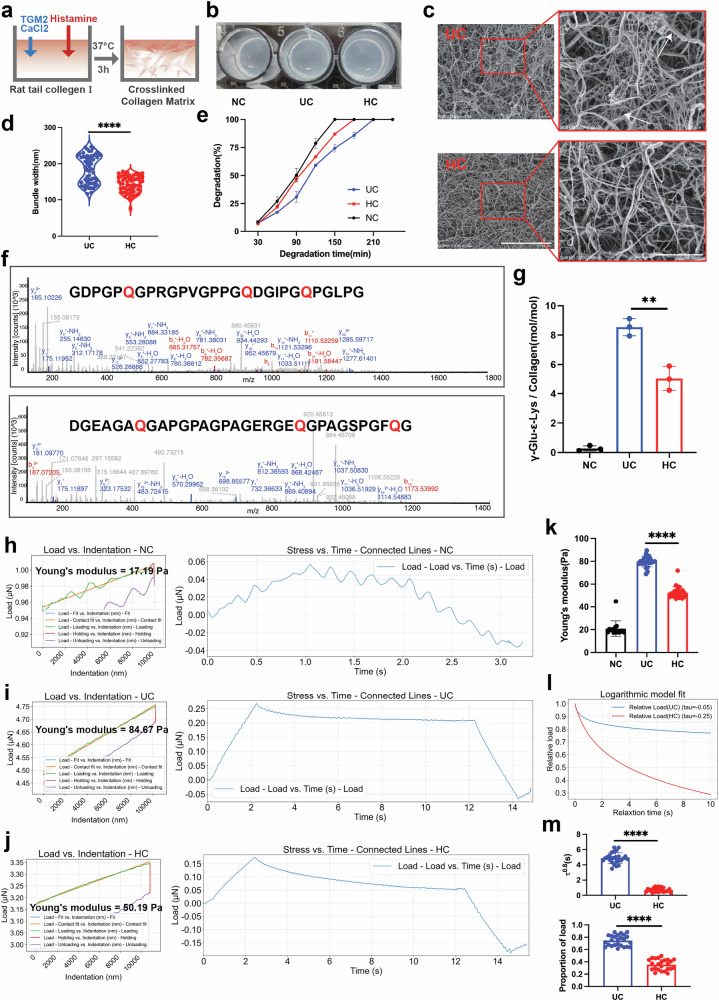
In vitro reconstruction and analysis of the TGM2-crosslinked collagen matrix. **a** Schematic illustration of collagen matrix construction. **b** Representative image of the collagen matrix crosslinked by TGM2. **c** SEM images of the ultrastructure of collagen fibrils (scale bars, left, 10 μm, right, 2 μm). **d** Quantification of alignment dimension after crosslinking (randomly selected, *n* = 50). **e** Degradation curves of collagen matrices digested with 0.08% collagenase I (*n* = 3). **f** LC-MS/MS detection of HC revealed multiple histaminylation modification sites. **g** AQMC quantification analysis of collagen crosslinking. HC showed significantly decreased γ-Glu-ε-Lys levels (*n* = 3). **h** Representative indentation and stress-relaxation curves of NC (Young’s modulus = 17.19 Pa). **i** Representative indentation and stress-relaxation curves of UC (Young’s modulus = 84.67 Pa). **j** Representative indentation and stress-relaxation curves of HC (Young’s modulus = 50.19 Pa). **k** Quantification of Young’s modulus of NC, UC and HC (*n* ≥ 16). **l** Representative fitted stress-relaxation curves of UC and HC. **m** Statistical comparison of relaxation time constants (τ^0.8^) and residual force of UC and HC (*n* ≥ 16). For all experiments, error bars represent the mean ± SD. *P < 0.05, **P < 0.01, ***P < 0.001, ****P < 0.0001

To assess the impact of histaminylation on collagen crosslinking properties, collagen matrices were prepared in vitro using rat tail type I collagen, recombinant TGM2, and histamine at an initial concentration of 20 µg/mL (Fig. 2a). There were no noticeable differences in the appearance between the collagen matrices with histaminylation (histaminylated collagen, HC) and without (unmodified collagen, UC) (Fig. 2b). However, scanning electron microscopy (SEM) data revealed that HC displayed markedly fewer fine crosslinks and a significantly reduced alignment dimension compared to UC (Fig. 2c, d). Moreover, collagenase I degradation assays showed that UC degraded most slowly, whereas HC underwent accelerated degradation, although still at a slower rate than natural self-assembled collagen matrix (NC) (Fig. 2e). Subsequently, LC-MS/MS analysis of histaminylation sites on the collagen matrix demonstrated a substantial number of modification sites on the α1 chain, whereas the α2 chain exhibited relatively fewer such sites (Fig. 2f, and Supplementary Dataset 3-4). The AQMC results also confirmed a significant reduction in γ-Glu-ε-Lys, indicating an inhibitory effect of TGM2-induced crosslinking in HC (Fig. 2g). In addition, we examined whether commercially obtained collagen exhibits histaminylation in the absence of any added reagents. We detected histaminylation at Q289 and Q974, suggesting that collagen histaminylation, as a covalent modification, may be stable and persist long-term in vivo (Supplementary Dataset 5).

BLASTP alignment revealed high conservation between rat and mouse type I collagen, enabling mapping of in vitro identified rat histaminylation sites to mouse sequences (Supplementary Fig. 6a). In the α1 chain, 10 of 31 Gln residues were histaminylated (Supplementary Table 1), while the α2 chain showed one modified Gln (Supplementary Data 2 and 4). Motif analysis using the MEME suite^22^ indicated that most modified residues occupied the Y position of the Gly-X-Y collagen motif without a clear X-position preference (Supplementary Fig. 6b). Comparative analysis with unmodified Gln sites using TwoSampleLogo^23^ confirmed an enrichment of the Gly-X-Y pattern around modified sites (Supplementary Fig. 6c), suggesting a sequence-based preference for collagen histaminylation. Furthermore, all detected modification sites are highly conserved within human type I collagen (Supplementary Table 2).

Based on our established collagen histaminylation model, we assessed the mechanical properties of NC, UC and HC. Among the matrices tested, NC exhibited the lowest Young’s modulus and, according to its stress-relaxation curve, displayed no evident viscoelastic behavior (Fig. 2h). In contrast, UC showed a significantly higher Young’s modulus than HC, and both crosslinked matrices demonstrated pronounced viscoelastic characteristics in their stress-relaxation curves (Fig. 2i, j). The Young’s modulus of the UC was approximately 80 Pa, which decreased to an average of approximately 50 Pa following histaminylation (Fig. 2k). Thus, these results demonstrated that histaminylation modulates collagen crosslinking at the microstructural level and reduces collagen matrix stiffness. Analysis of the normalized stress-relaxation curves revealed a marked alteration in the viscoelasticity of the histaminylated collagen matrix (Fig. 2l). Statistical analyzes of τ^0.8^ and the residual load similarly corroborated these findings (Fig. 2m). Furthermore, we evaluated the effect of a histamine concentration gradient (0, 5, 10, 40, and 80 µg/mL) on the mechanical properties of the collagen matrix. The results of mechanical testing showed that at approximately 20 µg/mL, the matrix stiffness reached its minimum, and no further reduction in Young’s modulus was observed with higher histamine concentrations (Supplementary Fig. 7). These findings suggest the existence of an upper limit to the number of histaminylation sites on collagen. Therefore, in subsequent experiments, a histamine concentration of 20 µg/mL will be employed for further investigation.

### Histaminylated collagen matrix markedly inhibits FMT in response to mechanical stretch

**Fig. 3:**
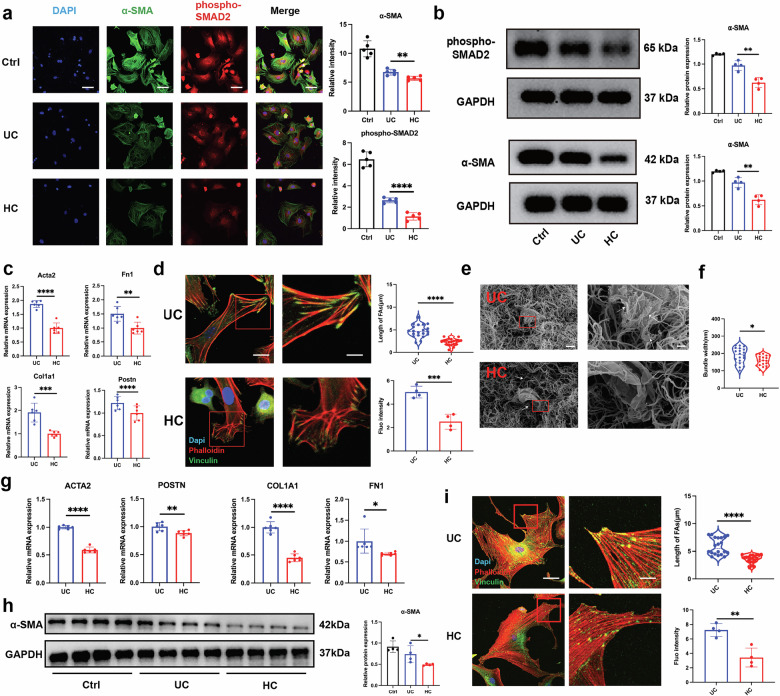
Fibroblasts cultured on UC and HC matrices under cyclic stretching combined with TGF-β stimulation exhibited marked differences in FMT and FA formation. **a** Representative and quantification of immunofluorescence staining of α-SMA and phospho-SMAD2 in NMCFs subjected to cyclic stretch on different matrices (*n* = 5) (Ctrl: uncoated soft-bottom plates, Young’s modulus = 1 kPa); scale bar, 50 µm. **b** Western blot analysis of the myofibroblast markers α-SMA and phospho-SMAD2 in NMCFs on different collagen matrices under stretch conditions (*n* = 4). **c** qRT-PCR data showing the expression of myofibroblast marker genes in NMCFs plated on UC and HC matrices (*n* = 6). **d** Representative and quantification of immunofluorescence staining of FA length (randomly selected, *n* ≥ 20) and intensity (*n* = 4) in NMCFs cultured on UC and HC matrices (scale bars, left 30 µm, right 10 µm). **e** SEM images showing the interaction between NMCFs and collagen matrices in the UC and HC groups (scale bars, left 2 µm, right 500 nm). **f** Quantification analysis of collagen alignment dimension of SEM images (*n* ≥ 20). **g** qRT-PCR data showing the expression of myofibroblast marker genes in human CF cell lines plated on UC and HC matrices (*n* = 6). **h** Western blot analysis of α-SMA in human CF cell lines on different collagen matrices under stretch conditions (*n* = 4). **i** Representative and quantification of immunofluorescence staining of FA length (randomly selected, *n* ≥ 20) and intensity (*n* = 4) in human CF cell lines cultured on UC and HC matrices (scale bars, left 50 µm, right 10 µm). For all experiments, error bars represent the mean ± SD. *P < 0.05, **P < 0.01, ***P < 0.001, ****P < 0.0001

To investigate the effects of the histaminylated collagen matrix on cardiac tissue cells, neonatal mouse cardiomyocytes (NMCMs) and fibroblasts (NMCFs) were used in the experiments. No observable effect of HC compared with UC on hypoxia-induced NMCM apoptosis was detected (Supplementary Fig. 8). Although previous studies have demonstrated that mechanical stretch can induce cardiomyocyte hypertrophy,^24^ the cell area analysis showed that NMCMs cultured on HC under stretch exhibited only a marginal reduction in spreading area compared with those on UC (Supplementary Fig. 9). Given these limited effects on NMCMs, we next sought to determine whether the mechanical properties of the collagen matrix primarily influence NMCFs. To predict whether the mechanical properties of collagen influence FMT under static conditions, we first employed the experimentally validated fibroblast-myofibroblast-collagen mathematical model (FMPCL).^25^ The results of mathematical modeling and western blot indicated that there was no significant decrease in the number of activated myofibroblasts after 24 h of simulation under static conditions (Supplementary Fig. 10). However, the most typical mechanical microenvironment encountered by cardiac tissue cells is not static but characterized by cyclic stretch. Therefore, we employed a cell stretcher and soft-bottom culture plates to test primary NMCFs grown on UC or HC. First, we observed that NMCFs cultured on soft matrices, whether on UC or on HC, proliferated rapidly under stretching (Supplementary Fig. 11a, c). After 24 h of cyclic stretching, β-actin exhibited marked polarization compared to nonstretched cells. This is consistent with previous studies,^26^ indicating that mechanical stretching promotes NMCF polarization and proliferation, even on a soft matrix (Supplementary Fig. 11b). However, the cell area post-stretching did not differ significantly between UC and HC matrices, suggesting that β-actin polarization is not affected by histaminylation of collagen (Supplementary Fig. 11d). We then evaluated the impact of HC versus UC on FMT after 24 h of cyclic stretch. The immunofluorescence and western blot results demonstrated that after 24 h of cyclic stretch, NMCFs cultured on HC exhibited significantly reduced FMT compared to those cultured on uncoated soft-bottom (Ctrl) dishes (Young’s modulus = 1 kPa) or on UC. Both α-SMA expression and TGF-β pathway activation, as measured by phosphorylated SMAD2 levels, were markedly downregulated (Fig. 3a, b). qRT-PCR data further confirmed that NMCFs cultured on HC exhibited significantly reduced expression of several myofibroblast markers, including *Postn*, *Fn1*, *Col1a1*, and *Acta2*, compared to those cultured on UC (Fig. 3c).

A previous study has shown that the extent of TGM2-mediated collagen crosslinking significantly influences the formation of focal adhesions (FAs).^27^ In this study, the results of immunofluorescence analysis revealed that myofibroblasts exhibited significantly shorter FAs with markedly reduced fluorescence intensity induced in HC compared to UC (Fig. 3d). Furthermore, SEM images showed a reduced bundle width of HC, and the myofibroblasts on the HC substrate displayed larger gaps between the cell membrane and the collagen substrate, along with a smoother interface at the cell-matrix contact area compared to those on the UC substrate (Fig. 3e, f). Consistent with previous studies, these results suggest that histamine can exert similar effects as TGM inhibitors by preventing collagen crosslinking, thereby reducing FA formation.^27^ Similarly, human CF cell lines exhibited the same trend, with HC treatment markedly attenuating FMT and FA formation (Fig. 3g–i).

### Matrix mechanical properties and collagen-associated integrin β1 regulate FMT following AMI via activation of PIEZO1 signaling

Mechanical signaling and integrins are essential components of cell-matrix communication. ITGB1, a collagen-binding integrin, coordinates with the mechanosensor PIEZO1 to regulate cellular functions. A previous study investigated a positive feedback loop between PIEZO1 and ITGB1 that promotes mechanotransduction.^28^ To explore their roles in FMT after AMI, we analyzed a human AMI scRNA-seq dataset.^29^ Clustering and cell type annotation were consistent with previous reports (Supplementary Fig. 12a). The results revealed that TGM2 is widely expressed among diverse cell types (Supplementary Fig. 2b). We applied the Gene Ontology Biological Process (GOBP) gene set “CELL_ADHESION_MEDIATED_BY_INTEGRIN” to score all cell populations. This analysis revealed widespread pathway activity across most cell types (albeit slightly weaker in cardiomyocytes) (Supplementary Fig. 12c) and demonstrated pronounced post-AMI upregulation in CFs, smooth muscle cells, myeloid cells, and adipocytes (Supplementary Fig. 12d). These results delineate the widespread cellular origins of TGM2 in the post-MI extracellular matrix and highlight the association of multiple cell populations with the mechanosensitive PIEZO1/ITGB1 axis. We then isolated CFs and performed reclustering analysis. Healthy donor CFs localized primarily to Cluster 0, which we designated quiescent CFs and the origin of the pseudotime trajectory (Supplementary Fig. 13a). Cluster 1, defined by elevated *ACTA2*, *POSTN*, *FN1*, and *COL1A1*, represented the myofibroblast state and was used as the terminal point (Supplementary Fig. 13b, c). Along this trajectory, the expression of myofibroblast markers increased substantially, accompanied by the upregulation of *ADAMTS2* and focal adhesion components (*VCL*, *PXN*, *ITGB1*), although the expression of *PIEZO1* itself remained minimally changed along the trajectory (Supplementary Fig. 13d). Co-expression analysis of proteins identified Module 3 as highly expressed in the myofibroblast subcluster, containing PIEZO1, COL1A1, COL1A2, POSTN and FN1 (Supplementary Fig. 13e, f). These bioinformatic analyzes indicate that PIEZO1 may play an important role in FMT following AMI.

**Fig. 4:**
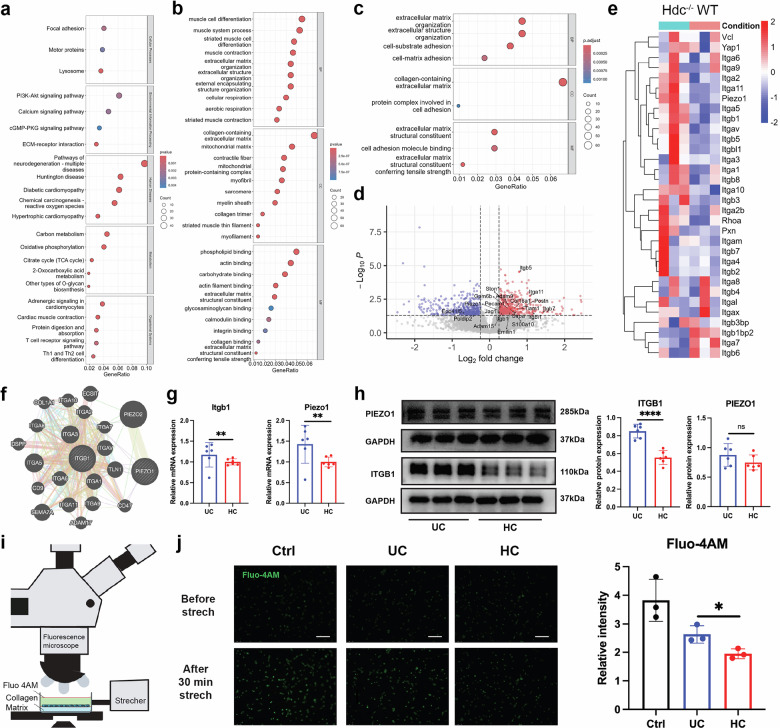
RNA-seq and experimental validation suggest an association between FMT and PIEZO1 signaling. **a** KEGG enrichment analysis of differentially expressed genes (DEGs) between *Hdc*^−/−^ and WT mouse hearts on day 7 post-AMI showed enrichment in focal adhesion and ECM-receptor interaction pathways. **b** Top 10 enriched GO pathways based on DEGs between *Hdc*^−/−^ and WT mouse hearts. **c** Enriched pathways related to ECM and cell adhesion in further GO analysis. **d** Volcano plot highlighting DEGs from the GO cell-matrix gene set. **e** Expression profiles of integrins, FA markers, and *Piezo1* in *Hdc*^−/−^ and WT mouse hearts on day 7 post-AMI demonstrated the upregulation of multiple integrins in the *Hdc*^−/−^ group. **f** Protein‒protein interaction prediction analysis of PIEZO1 and integrins by the GeneMANIA (https://genemania.org/) dataset. **g** qRT-PCR analysis of *Itgb1* and *Piezo1* mRNA levels in NMCFs cultured on UC and HC collagen matrices (*n* = 6). **h** Western blot analysis of ITGB1 and PIEZO1 protein levels in NMCFs on UC and HC matrices (*n* = 6). **i** Schematic representation of the uniaxial cyclic stretch setup under fluorescence microscopy. **j** Representative images and quantification of Fluo-4AM staining after 30 min of cyclic uniaxial stretch in NMCFs cultured on UC, HC, and control groups (Ctrl: uncoated soft-bottom plates, Young’s modulus = 1 kPa; *n* = 3; scale bar, 200 µm). For all experiments, error bars represent the mean ± SD. *P < 0.05, **P < 0.01, ***P < 0.001, ****P < 0.0001

We next examined the differentially expressed genes from bulk RNA-seq of cardiac tissue collected in *Hdc*^−/−^ versus WT mice on day 7 post AMI. Kyoto Encyclopedia of Genes and Genomes (KEGG) pathway enrichment analysis revealed that these differentially expressed genes were predominantly enriched in the “Focal Adhesion” and “ECM-Receptor Interaction” pathways (Fig. 4a). Gene Ontology (GO) enrichment analysis further demonstrated significant enrichment of several ECM-related terms, including “collagen-containing extracellular matrix” and “integrin binding” (Fig. 4b, c). In the cardiac tissue of *Hdc*^−/−^ mice 7 days post AMI, several integrins were upregulated, namely, collagen-binding integrin *Itgb1*, as well as immune cell adhesion-associated *Itgb5*, *Itgb7*, and *Itga11* (Fig. 4d). Moreover, *Piezo1* itself showed a very modest increase in expression, and its downstream effectors *Yap1* and *Rhoa* trended upward, but the increases were not statistically significant (Fig. 4e). GeneMANIA^30^-based protein interaction network analysis indicated that PIEZO1 is functionally linked to multiple integrins (Fig. 4f). To verify the presence of these changes under UC and HC conditions in vitro, we performed qRT-PCR and western blot assays. The results confirmed that the expression levels of *Piezo1* and *Itgb1* mRNA were significantly reduced in cells cultured on HC compared to those on UC (Fig. 4g). Consistently, Western blot analysis confirmed a significant reduction in ITGB1 protein in CFs subjected to mechanical stretching on HC compared to those on UC, but no significant change in PIEZO1 protein (Fig. 4h). Together, these results suggest that PIEZO1 exhibits relatively stable protein expression. Nevertheless, as a mechanosensitive calcium channel, neither its transcriptional levels nor its protein abundance reliably represent its functional state.^31^ To evaluate PIEZO1-mediated intracellular calcium influx, we constructed a system integrating fluorescence microscopy with a uniaxial cell stretcher and employed Fluo-4AM as a calcium indicator (Fig. 4i, and Supplementary Movie 1). Fluorescence imaging showed that NMCFs cultured on uncoated soft substrates (Young’s modulus = 1 kPa), UC, and HC all exhibited increased intracellular calcium fluorescence intensity after 30 min of uniaxial stretching compared to the unstretched condition. However, the magnitude of this increase progressively declined across the three substrates (Fig. 4j). These findings demonstrate that HC suppresses the expression of *Piezo1* and *Itgb1* in stretched NMCFs and inhibits PIEZO1 channel-mediated calcium influx.

### HC inhibits FMT by downregulating the mechanosensitive PIEZO1/ITGB1 signaling axis

**Fig. 5:**
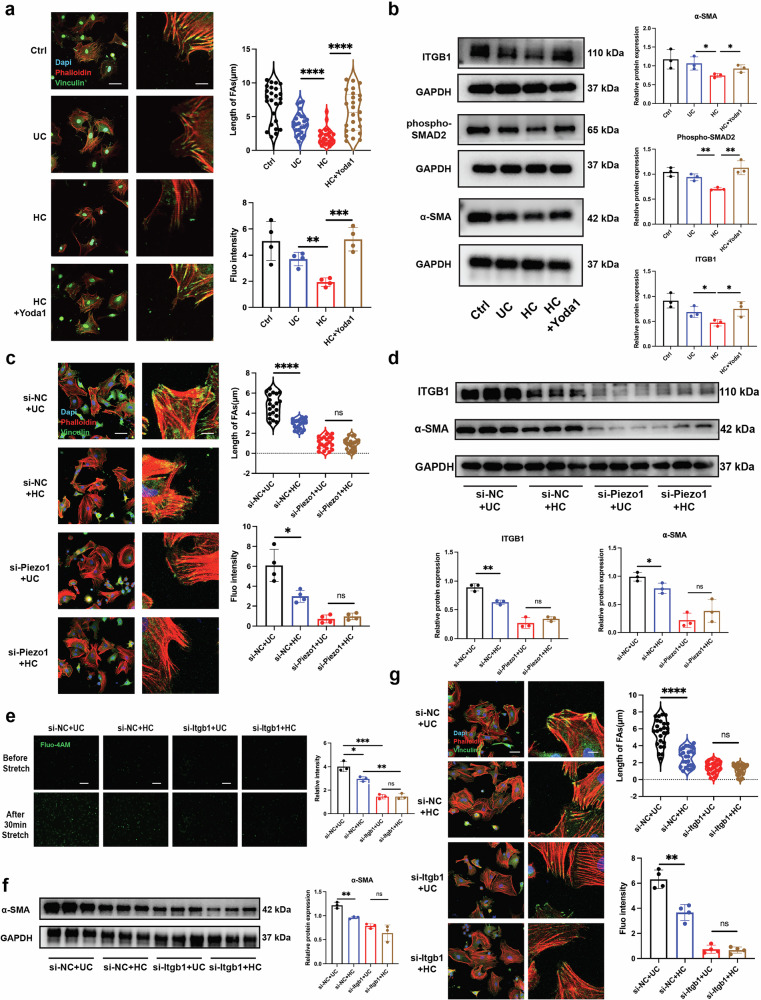
PIEZO1/ITGB1 modulates mechanotransduction and FMT in NMCFs cultured on different collagen matrices. **a** Immunofluorescence images and quantification of FA length (randomly selected, *n* ≥ 20) and intensity (*n* = 4) in NMCFs cultured on UC, HC, and HC supplemented with the PIEZO1 agonist Yoda (scale bar, left 50 µm, right 10 µm). **b** Western blot analysis of ITGB1, phospho-SMAD2, and α-SMA protein levels in NMCFs cultured on UC, HC, and HC+Yoda1 (*n* = 3). **c** Immunofluorescence analysis of FA length (randomly selected, *n* ≥ 20) and intensity (*n* = 4) in NMCFs cultured on UC and HC after siRNA-mediated knockdown of *Piezo1* (scale bar, left 50 µm, right 10 µm). **d** Western blot analysis of ITGB1 and α-SMA protein expression in NMCFs cultured on UC and HC after *Piezo1* knockdown by siRNA (*n* = 3). **e** Representative images and quantification analysis of Fluo-4AM staining after 30 min of cyclic uniaxial stretch in *Itgb1* knockdown NMCFs cultured on UC and HC (*n* = 3; scale bar, 200 µm). **f** Western blot analysis of α-SMA protein expression in NMCFs cultured on UC and HC after *Itgb1* knockdown by siRNA (*n* = 3). **g** Immunofluorescence analysis of FA length (randomly selected, *n* ≥ 20) and intensity (*n* = 4) in NMCFs cultured on UC and HC after siRNA-mediated knockdown of *Itgb1* (scale bar, left 50 µm, right 10 µm). For all experiments, error bars represent the mean ± SD. *P < 0.05, **P < 0.01, ***P < 0.001, ****P < 0.0001

We next treated stretched NMCFs cultured on HC with the PIEZO1 agonist Yoda1. Immunofluorescence data showed that Yoda1 markedly increased FA formation (Fig. 5a), while western blot data showed a parallel rise in ITGB1 protein and a strengthened FMT, as evidenced by upregulated α-SMA and phosphorylated SMAD2 (Fig. 5b). In addition, we silenced *Piezo1* in primary NMCFs with siRNA and subjected both si-NC and si-*Piezo1* cells to mechanical stretching on UC and HC substrates (Supplementary Fig. 14). FA formation was markedly inhibited in si-*Piezo1* NMCFs on both matrices, abolishing the difference previously observed in HC (Fig. 5c). Western blot data further showed that ITGB1 and α-SMA levels were significantly decreased in si-*Piezo1* cells on both UC and HC, with no appreciable difference between the two substrates (Fig. 5d).

We subsequently conducted knockdown experiments targeting *Itgb1*. *Itgb1* knockdown further diminished PIEZO1-mediated stretch-evoked Ca^2+^ influx and eliminated the differences observed between the UC and HC conditions (Fig. 5e). Consistent with the phenotype observed upon *Piezo1* knockdown, *Itgb1* knockdown markedly attenuated FMT and FA formation, and no significant UC-HC differences remained (Fig. 5f, g). Similarly, knockdown of *Itga2*, the α-subunit that heterodimerizes with *Itgb1* to form the collagen-binding integrin receptor, resulted in comparable phenotypes (Supplementary Fig. 15). Collectively, these data demonstrate that HC suppresses PIEZO1-dependent Ca^2+^ entry and ITGB1 expression, leading to a decrease in FMT. PIEZO1 and ITGB1 might participate in a reinforcing positive feedback loop.

### Histamine-delivering hydrogel significantly attenuates myocardial fibrosis in *Hdc*^−/−^ mice post-AMI

**Fig. 6:**
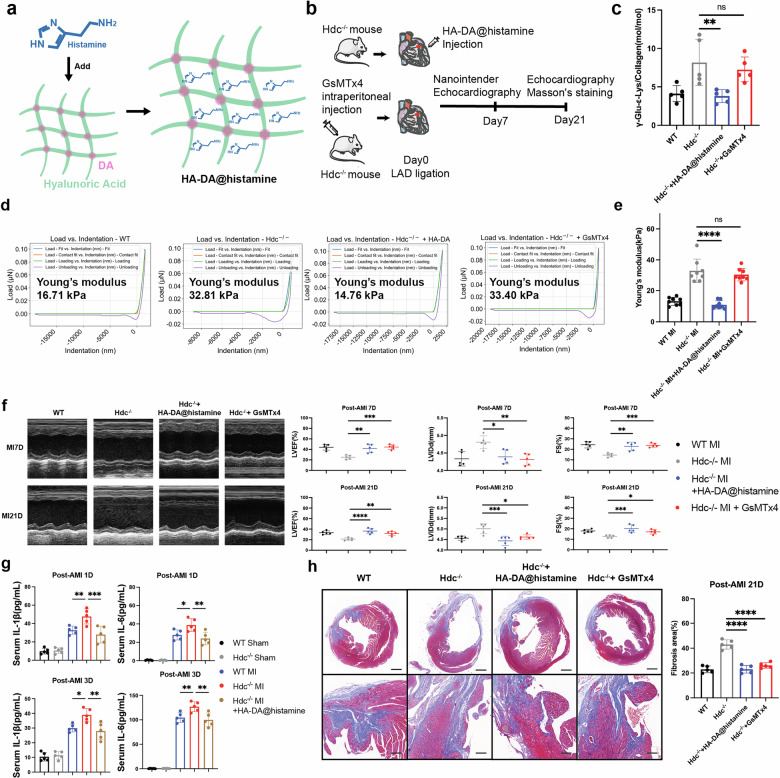
Supplementation with histamine or inhibition of PIEZO1 significantly attenuates myocardial fibrosis in *Hdc*^−/−^ mice post-AMI. **a** Construction of the HA-DA@histamine hydrogel. **b** Schematic of the *Hdc*^−/−^ mouse AMI model treated with HA-DA@histamine and GsMTx4. **c** AQMC results of γ-Glu-ε-Lys in the cardiac tissue of WT, *Hdc*^−/−^, *Hdc*^−/−^ treated with HA-DA@histamine and *Hdc*^−/−^ treated with GsMTx4 mice 7 days post-AMI (*n* = 5). **d** Representative indentation curves show the difference between WT and *Hdc*^−/−^ mice treated with HA-DA@histamine and GsMTx4. **e** Quantitative analysis of Young’s modulus in the scar region of WT mice, *Hdc*^−/−^ mice, *Hdc*^−/−^ mice treated with HA-DA@histamine and *Hdc*^−/−^ treated with GsMTx4 mice 7 days post-AMI (*n* = 8). **f** Representative results and quantitative analysis of echocardiography on day 7 and day 21 post-AMI (*n* = 5). **g** ELISA results show the levels of IL-1β and IL-6 in the serum on day 1 and day 3 post-AMI (*n* = 6). **h** Representative results of Masson staining and quantitative analysis of cardiac fibrosis (*n* = 5; scale bar, top 1000 μm, bottom 250 μm). For all experiments, error bars represent the mean ± SD. *P < 0.05, **P < 0.01, ***P < 0.001, ****P < 0.0001

We previously engineered a dopamine-crosslinked hyaluronan hydrogel (HA-DA@Histamine) capable of delivering histamine in a sustained manner (Fig. 6a).^32^ To investigate whether supplementing exogenous histamine could affect collagen histaminylation and cardiac fibrosis in *Hdc*^−/−^ mice post-AMI, HA-DA@histamine was administered via injection at the time of infarction induction (Fig. 6b). The PIEZO1 inhibitor GsMTx4 has been shown to confer cardioprotective effects in WT mice post-AMI,^33^ and we likewise rescued AMI in *Hdc*^−/−^ mice by blocking PIEZO1 channels. The results demonstrated that HA-DA@histamine significantly reduced cardiac γ-Glu-ε-Lys levels compared with the *Hdc*^−/−^ control group post-AMI, whereas no such significant trend was observed in the GsMTx4-treated group (Fig. 6c). Measurement of the Young’s modulus in the infarcted region revealed a significant reduction in stiffness in the HA-DA@histamine group compared with the *Hdc*^−/−^ control group, whereas the GsMTx4-treated group showed no significant difference from the *Hdc*^−/−^ control group (Fig. 6d, e). The results of echocardiographic analysis demonstrated a significant increase in LVEF and FS in both the HA-DA@histamine group and the GsMTx4 group compared with the control group, with a significant decrease in LVIDd (Fig. 6f). Remarkably, localized hydrogel application significantly reduced the systemic and local inflammation and the accompanying immune cell infiltration induced by histamine deficiency (Fig. 6g, and Supplementary Fig. 16). Masson’s staining confirmed that the fibrosis area was significantly reduced in both the HA-DA@histamine and GsMTx4 groups compared with the control group (Fig. 6h). The above results indicate that histamine supplementation via HA-DA@histamine significantly attenuates excessive collagen crosslinking in *Hdc*^−/−^ mice, thereby restoring cardiac function and reducing myocardial fibrosis post-AMI. Meanwhile, although Piezo1 blockade could not prevent excessive collagen crosslinking caused by histamine deficiency, it alleviated cardiac dysfunction and myocardial fibrosis in *Hdc*^−/−^ mice post-AMI.

## Discussion

Fibrosis is a major pathological feature of post-AMI cardiac remodeling, yet its underlying mechanisms remain a research challenge.^1^ The ECM is an important component of the cardiac microenvironment, and changes in its physicochemical properties can regulate cellular function through mechanical signal transduction. Cardiac cell-matrix interactions and mechanotransduction by FAs after myocardial ischemic injury can regulate FMT, but their mechanisms urgently need to be elucidated.^3^ In the current study, we first identified histaminylation, a previously unrecognized PTM, in cardiac type I collagen fibers after AMI. Using histamine-deficient *Hdc*^−/−^ mice and an in vitro collagen histaminylation model, we demonstrated that histaminylation may alter matrix mechanical properties, attenuate TGM2-mediated crosslinking by competing for Gln residues, and suppress FMT while downregulating the mechanosensitive PIEZO1/ITGB1 signaling pathway. In addition, this study revealed new interactions among *Hdc*-expressing myeloid cells, CFs and the extracellular microenvironment. A new strategy has been proposed to inhibit myocardial fibrosis and protect injured cardiomyocytes after ischemic injury by histaminylation.

Similar to other tissues and organs, the cardiac connective tissue is predominantly composed of fibrillar collagens and associated proteoglycans. Collagen types I and III are the major constituents, while type V collagen is present in smaller amounts.^34^ In cardiac tissues, collagen crosslinking has been primarily categorized into three types, but research on TGM2-mediated collagen crosslinking in cardiac fibrosis remains limited.^5^ A recent study demonstrated that inhibition of TGM2 attenuates cardiomyocyte apoptosis and cardiac fibrosis following AMI, potentially through downregulation of the NF-κB signaling pathway. However, the study did not further investigate the role of TGM2 in collagen crosslinking.^35^ In this study, we focused on TGM2-mediated collagen crosslinking following AMI. A marked upregulation of TGM2 expression was observed during the early phase post-AMI. In line with previous studies, we employed the AQMC assay to assess cardiac tissue and observed a significant increase in γ-Glu-ε-Lys on day 7 post-AMI. This finding supports the critical roles of TGM2-mediated collagen crosslinking in scar formation and myocardial fibrosis following AMI.

Collagen matrix formation occurs in two stages: intracellularly, procollagen, comprising three peptide chains is synthesized in an unprocessed form, which is then secreted into the extracellular space where N- and C-terminal propeptides are cleaved. Subsequent crosslinking is mediated by LOX, AGEs, and TGM2.^36^ Interestingly, although only a few studies have reported that monoamine modification inhibits protein crosslinking, such as the histaminylation of fibrinogen^37^ and the dopaminylation of tau protein,^38^ its effect on collagen crosslinking, which represents the major component of the ECM, remains largely unexplored. Therefore, we hypothesize that the histaminylation of collagen, whether occurring intracellularly or extracellularly, primarily affects the final crosslinking step mediated by TGM2. In this study, the abnormally shortened D-period spacing observed in *Hdc*^−/−^ mice, along with the increased viscoelasticity of HC in vitro, supports this notion. Excessive collagen crosslinking leads to an elevated Young’s modulus of type I collagen, which reduces D-period extensibility during the so-called “heel stage” of tensile loading (Fig. 1e). Additionally, the stress-relaxation behavior of the excessively crosslinked collagen matrix is altered compared to that of normally crosslinked collagen.^27,39^

In addition to structural alterations affecting the mechanical properties of collagen, certain specific domains within the collagen molecule may also be influenced by histaminylation. The N-terminal cleavage of procollagen, primarily mediated by members of the ADAMTS family (notably ADAMTS2, 3, and 14), is essential for proper collagen crosslinking. Impaired cleavage can disrupt normal crosslink formation, leading to disorganized collagen architecture.^40^ The cleavage of procollagen by ADAMTS enzymes relies on recognition of specific N-terminal sequences, such as the serine and glutamine residues within the 158-165 region of the type I collagen α1 chain (NFASQMSY).^41^ In this study, we identified histaminylation at ADAMTS recognition motifs on both the α1 and α2 chains of type I collagen (Supplementary Table S1, and Supplementary Dataset 1, 4). However, whether histaminylation affects the N-terminal processing of procollagen remains to be further investigated.

Consistent with previous studies, our findings demonstrate that crosslinked collagen exhibits pronounced viscoelastic and stress-relaxation properties and that the degree of crosslinking significantly influences the mechanical characteristics of the collagen matrix, including its stiffness and viscoelasticity.^42^ Fibroblasts are mechanosensitive and exhibit “durotaxis,” a tendency to migrate toward stiffer substrates. Matrix stiffness plays a crucial role in regulating the proliferation and differentiation of fibroblasts.^43^ As the principal site of cell-matrix interactions, FAs also serve as key mechanosensors that respond to changes in the extracellular mechanical environment, and their formation and maturation are regulated by mechanical cues.^44^ Prior studies have shown that TGM2 knockdown suppresses focal adhesion kinase (FAK) activation, thereby reducing FA formation.^45^ Another study reported that the TGM2-crosslinked collagen matrix significantly promoted FA formation compared to the noncrosslinked matrix.^27^ However, previous studies have focused solely on the crosslinking function of TGM2 in collagen while overlooking its role in monoaminylation. In this study, histamine acted similarly to a TGM2 inhibitor, with histaminylation blocking collagen crosslinking by occupying Gln residues, thereby inhibiting FA formation.

Was initially discovered as a protein that interacts with integrins and contributes to the formation of cell adhesions.^46^ *Piezo1* knockdown has been reported to reduce integrin expression, including *Itgb1*, in various cell types, such as human preputial fibroblasts, human mammary epithelial cells, and human umbilical vein endothelial cells.^47^ However, to our knowledge, no such findings have been reported in CFs. PIEZO1 is a mechanosensitive calcium channel that partially localizes to mature FAs.^48^ Knockdown of *Piezo1* leads to reduced FA formation,^49^ and FAs located near PIEZO1 can induce its activation and trigger calcium influx,^50^ suggesting a bidirectional relationship between PIEZO1 and integrins. In the current study, we first observed the upregulation of both *Itgb1* and *Piezo1* at the mRNA level in the hearts of *Hdc*^−/−^mice, indicating that histamine deficiency may result in the activation of the PIEZO1/ITGB1 axis. PIEZO1 likely senses changes in matrix stiffness and viscoelasticity, whereas ITGB1 may be influenced not only by mechanical cues but also by collagen crosslinking and structural alterations, as previously described. Therefore, we propose that PIEZO1 and ITGB1 are not sequentially regulated but rather co-activated in a histamine-deficient collagen matrix, working synergistically to mediate the cellular response.

Unlike other organs, the heart is subjected to continuous mechanical strain from the earliest stages of morphogenesis.^51^ Cyclical stretch, as one of the key mechanical features of the heart, has been shown to activate CFs.^52^ Another study demonstrated that cyclical stretch can activate PIEZO1, thereby promoting fibroblast activation.^53^ However, how the ECM regulates CF proliferation in the heart’s complex mechanical environment remains largely unclear. In this study, we simulated cardiac mechanical conditions using a cyclically stretched soft substrate and found that, consistent with previous reports,^26^ cells exhibited prominent formation and extension of stress fibers on a soft matrix. Notably, the effect of histaminylation was significant only under dynamic stretching conditions. Interestingly, in line with the dynamic nature of organs such as the heart, our previous work also showed that *Hdc*^−/−^ mice lacking histamine exhibited more severe tissue damage in a hindlimb ischemia model.^32^ These findings suggest that histaminylation may play a unique role in dynamic, mechanically active tissues such as myocardium, skeletal muscle, skin, or cartilage.

Several studies targeting TGM2 inhibition have explored potential strategies to intervene in various diseases. Some studies have focused on inhibiting the intracellular signaling pathways regulated by TGM2. For instance, GK921 has been used to suppress TGM2 activity, thereby inhibiting the mesenchymal transition of glioma stem cells through the modulation of the C/EBPβ signaling pathway.^54^ Other studies have focused on the role of TGM2 in collagen crosslinking. Notably, inhibition of TGM2 by cystamine has been shown to reduce calcification of aortic rings in chronic kidney diseases by blocking ECM crosslinking.^55^ Our study indicates that histaminylation does not completely block collagen crosslinking. This suggests that, compared to fully inhibiting TGM2 activity, histaminylation may serve as a more “modest” regulatory mechanism by occupying Gln residues to partially suppress TGM2 function. In this way, it allows for the necessary collagen crosslinking required for tissue repair while fine-tuning the mechanical properties of the ECM. In this study, we focused specifically on collagen; however, TGM2 is also known to catalyze the crosslinking of various other ECM proteins, such as fibronectin^55^ and glycoproteins.^56^ Although we successfully engineered in vitro histaminylated collagen matrices, they do not fully replicate the mechanical stiffness of the native cardiac ECM. Our findings indicate that both UC (approximately 80 Pa) and HC (approximately 50 Pa) still represent relatively very soft substrates compared to normal cardiac tissue (approximately 10 kPa). This may also partially explain why we failed to observe a significant difference in cardiomyocyte apoptosis or hypertrophy between the UC and HC groups (Supplementary Figs. 8 and 9), as the mechanical alterations of the in vitro collagen matrix might not have reached the threshold required to impact cardiomyocyte apoptosis. Nevertheless, at the level of cardiac tissue, our study revealed that histamine deficiency also led to significant changes in Young’s modulus, suggesting that the change in histaminylation plays a critical role in regulating the overall mechanical properties of cardiac ECM. In theory, histaminylation may also regulate the crosslinking of other ECM proteins by occupying Gln residues. Nevertheless, further investigations are required to confirm this possibility and to elucidate its physiological relevance.

Histamine is a multifunctional monoamine that exerts diverse receptor-mediated effects after myocardial infarction, including the regulation of cardiomyocyte apoptosis,^15^ fibroblast proliferation,^13^ and macrophage-myofibroblast transition.^17^ It also plays an important regulatory role in immune cells, including modulating macrophage reprogramming^32^ and neutrophil NETosis.^14^ In contrast, histaminylation represents an emerging PTM whose in vivo functions remain poorly understood, particularly within the ECM. Dissecting receptor-independent roles of histaminylation is technically challenging. For intracellular targets, cell type-specific deletion of TGM2 can abolish monoaminylation^57^; however, ECM-resident TGM2 originates from multiple cellular sources (Supplementary Fig. 11b), and global deletion of this multifunctional enzyme disrupts numerous physiological pathways, introducing substantial confounding effects. Likewise, site-directed mutagenesis of collagen is impractical, as multiple histaminylated glutamine residues were identified, and these residues are also required for physiological crosslink formation. Given these constraints, histamine-deficient *Hdc*^−/−^ mice currently represent the most feasible model for studying collagen histaminylation in vivo, although receptor-dependent effects cannot be fully excluded. Nevertheless, our AQMC analyzes clearly demonstrate abnormal and excessive collagen crosslinking in *Hdc*^−/−^ mice, supporting a receptor-independent contribution of histaminylation to post-AMI ECM remodeling. Direct administration of histamine may lead to pronounced fluctuations in local or systemic histamine levels, as well as rapid metabolic degradation. Our histamine-releasing hydrogel formulation may mitigate these issues to some extent.^32^ However, selectively modulating monoaminylation may require alternative molecular strategies. One potential approach involves the use of 1-methylhistamine, the major product of histamine inactivation by HNMT. Although 1-methylhistamine lacks the ability to activate classical histamine receptors, it retains the primary amine necessary for monoaminylation chemistry and may therefore preserve modifying activity without inducing receptor signaling.^58^ Whether such analogs can selectively influence ECM histaminylation in vivo warrants further investigation. Reducing the degradation of 1-methylhistamine through MAO-B inhibitors (such as isatin) may also be a potential strategy, and this approach has shown certain protective effects in the infarcted mice hearts in a recent study.^59^

This study has several avenues for future investigation. First, we were unable to quantitatively assess the extent of histaminylation, largely due to the lack of specific detection probes. Moreover, type I collagen contains multiple Gln residues susceptible to histaminylation, and the resulting peptides are heterogeneous, posing significant challenges for the development of specific antibodies. Second, this study focused solely on the effects of histaminylation on the ECM, without addressing its potential roles on intracellular or other extracellular proteins, despite TGM2 being a broadly active enzyme. Finally, although our bioinformatic analyzes identified the PIEZO1/ITGB1 axis as a downstream pathway affected by histaminylated collagen, other mechanoreceptors and signaling pathways were not explored in this study.

Our study identified histaminylation as a previously unrecognized PTM of cardiac type I collagen after AMI. We demonstrated that histaminylation modulates matrix mechanics, competes with TGM2 for Gln residues to attenuate crosslinking, and promotes FMT via PIEZO1/ITGB1 signaling. Moreover, the findings of this study reveal novel interactions among *Hdc*-expressing myeloid cells, CFs and the extracellular matrix, suggesting histaminylation as a potential therapeutic strategy for myocardial fibrosis and cardiomyocyte protection after ischemic injury.

## Materials and methods

### Mice

*Hdc*-GFP and Hdc^−/−^ mouse strains were kindly provided by Professor Timothy C. Wang of Columbia University, and both were maintained on a BALB/c genetic background. WT BALB/c controls were procured from the Department of Laboratory Animal Science at Fudan University (Supplementary Table S2). All animals were housed under specific pathogen-free conditions in Fudan University’s Animal Care Facility. Male mice at 8 weeks of age were used for in vivo experiments. Experimental procedures complied with institutional guidelines for laboratory animal care and were approved by the Fudan University Committee on the Ethics of Animal Experiments (Approval No. SYXK 2021-0022).

### Cardiac collagen extraction

Cardiac crude collagen was extracted from mouse hearts 7 days post AMI following a previously described protocol.^19^ Briefly, heart tissues were minced, washed with 10% NaCl, homogenized, and centrifuged at 10,000 × *g* for 30 min at 4 °C. Pellets were washed, defatted with ethanol, and filtered. Collagen was extracted by gentle shaking in 50 mM acetic acid with 5 mg/mL pepsin at 4 °C, followed by precipitation with 2 M NaCl. The precipitate was dissolved in distilled water, neutralized with 10 mM phosphate buffer (pH 8.0), incubated overnight at 25 °C, and re-centrifuged. The final collagen pellet was redissolved in 5 mM acetic acid.

### In vitro collagen matrix construction

All reagents were pre-chilled on ice for 1 h. Rat tail type I collagen (Corning) was diluted to 2 mg/mL according to the concentration indicated on the bottle. A 5 mg/mL histamine hydrochloride (Sigma Aldrich) solution was prepared using phosphate-buffered saline (PBS), while recombinant guinea pig liver TGM2 (Zedira) was diluted in PBS to a concentration of 10 μ/mL. A 1 M CaCl_2_ (Macklin) solution was prepared using distilled water. Reaction mixtures were prepared on ice according to experimental needs with the following final concentrations: collagen, 2 mg/mL; CaCl_2_, 5 mM; histamine hydrochloride, 20 μg/mL; TGM2, 0.2 μ/mL. After mixing, Tris base was added to adjust the pH to 7.2–7.4. The reaction mixtures were transferred to designated molds or plates and incubated at 37 °C for 3 h. After crosslinking, the samples were washed three times with a small volume of PBS and stored at 4 °C in PBS.

### LC/MS-MS detection of histaminylation

Mobile phase A consisted of 100% water with 0.1% formic acid, and phase B contained 80% acetonitrile with 0.1% formic acid. Lyophilized samples were dissolved in 10 µL of phase A, and centrifuged (14,000 × *g*, 20 min, 4 °C), and 1 µg of supernatant was injected for LC-MS analysis. Protein detection was carried out on an ORBITRAP ECLIPSE mass spectrometer (QL-HPLC-100*15 column) with a FAIMS Pro^TM^ Interface (CV: −45/−65 V, 1 s switching). The Nanospray Flex™ (NSI) source was set at 2.0 kV and 320 °C. Data were acquired in DDA mode with full MS scans (*m*/z 350–1500, resolution 120,000, AGC 4 × 10^5^, injection time 50 ms) and MS/MS scans (“Top Speed” mode, resolution 15,000 at *m*/*z* 200, AGC 5 × 10^4^, injection time 22 ms, 33% collision energy). Raw data (.raw) were processed using Proteome Discoverer 2.4 against the target database, with trypsin as the protease (≤ 3 missed cleavages). Carbamidomethylation (C) was set as static, while oxidation (M), N-terminal acetylation, and C_5_H_6_N_2_ modification (Q) were dynamic. Mass tolerances were ±15ppm for precursors and ± 0.02 Da for fragments.

### Mechanical property determination

Nanoindentation measurements were performed using a Piuma Nanoindenter (Optics11, Netherlands) in displacement-controlled mode for cardiac tissue and force-controlled mode for collagen matrices. Cardiac tissue processing and post-AMI scar localization were performed as described previously.^60^ Briefly, mouse hearts 7 days after AMI were isolated and embedded in OCT and cryosectioned, and scar regions were identified by dense type I collagen immunofluorescence signals, which served as a guide for nanoindenter-based indentation testing. The probes used had a cantilever stiffness ranging from 0.46 to 0.5 N/m and were equipped with a spherical tip with a 27.5 μm radius. During measurements, both the cantilever and the sample were immersed in PBS and stabilized using waterproof adhesive to minimize capillary force-related artifacts. Raw data were initially processed using Optics11 Dataviewer software and further analyzed and visualized using custom Python scripts.

### Cyclic stretch of cells

NMCFs and human CFs cell lines were seeded at a density of 1 × 10^5^ cells/cm^2^ onto flexible silicon membranes (FlexCell) coated with UC or HC collagen and subjected to uniaxial, pulsatile stretch (110%, 1.5 Hz) for 24 h using the FX-6000T Tension System (FlexCell). Cells seeded on FlexCell culture plates without collagen coating served as controls. For Fluo-4 AM fluorescence imaging, NMCFs were plated on UC- or HC-coated flexible silicon membrane bottom chambers (Cell & Force), with uncoated membranes as controls. After 24 h of culture, the medium was removed, and cells were stained with 1 μM Fluo-4 AM for 60 min, followed by three washes with HBSS. NMCFs were then incubated at 37 °C for 30 min. Subsequently, a Beatle stretching system (Cell & Force) was used to apply uniaxial stretch (1 cm, 1.5 Hz) for 30 min under fluorescence microscopy, and intracellular calcium levels were monitored at an excitation.

### SEM and TEM

Tissue samples were trimmed to ≤1mm^3^ to minimize mechanical damage and fixed in EM fixative at 4 °C for 2–4 h. Collagen matrices seeded with cells were similarly cut and fixed. All samples were washed three times with 0.1 M PBS (pH 7.4, 15 min each), post-fixed in 1% osmium tetroxide (RT, 2 h), and rewashed in PBS. Dehydration was performed using a graded ethanol series (50%–100%, 15 min each). Samples were infiltrated overnight with a 1:1 mixture of acetone and Epon 812, followed by pure resin infiltration and embedding at 60 °C for 48 h. Ultrathin sections (60–80 nm) were cut with an ultramicrotome, stained with 2% uranyl acetate and lead citrate (15 min each), and air-dried. Transmission electron microscopy (TEM, FEI Tecnai G2 20 TWIN) was used to examine ultrastructure. Where applicable, scanning electron microscopy (SEM, HITACHI SU8100) was employed in parallel to visualize surface morphology.

### Statistical analysis

Data are presented as mean ± standard deviation. Statistical analysis was performed using GraphPad Prism 9. Student’s *t*-test was used for two-group comparisons, and one-way ANOVA for multiple group comparisons. A *p*-value below 0.05 was considered statistically significant.

More detailed methodological information is provided in the Supplementary Materials.

## Supplementary information

Supplementary Materials for Histaminylation alters collagen matrix mechanics and attenuates cardiac fibrosis post-myocardial infarction via mechanotransduction signaling axis

Movie S1

Data S1

Data S2

Data S3

Data S4

Data S5

Data S6

## Data Availability

ScRNA-seq datasets used in this study are, respectively, stored in GEO (GSE163129) and cellxgene (https://cellxgene.cziscience.com/collections/8191c283-0816-424b-9b61-c3e1d6258a77). Bulk RNA-seq data was stored in CNCB (CRA038888). All the other data necessary to understand and evaluate the conclusions of this study are provided in the supplementary information.
