# Supplementary material for: Histaminylation alters collagen matrix mechanics and attenuates cardiac fibrosis post-myocardial infarction via mechanotransduction signaling axis
Source: Signal Transduct Target Ther. 2026 Jun 11;11:229. doi: 10.1038/s41392-026-02721-5 (PMC13254370; doi:10.1038/s41392-026-02721-5)
Supplement: Supplementary file 1 — Supplementary Materials for Histaminylation alters collagen matrix mechanics and attenuates cardiac fibrosis post-myocardial infarction via mechanotransduction signaling axis [file 41392_2026_2721_MOESM1_ESM.docx]

Supplementary Materials for

Histaminylation alters collagen matrix mechanics and attenuates cardiac fibrosis post-myocardial infarction via mechanotransduction signaling axis

Jianfu Zhu,† Dili Sun,† Gaofeng Zeng,† Kehan Wu, Xiyang Yang, Xiaowei Zhu, Diyaerjiang Aierken, Suling Ding, Xiangfei Wang,* Junbo Ge,* and Xiangdong Yang*

Correspondence to:Xiangfei Wang*: wang.xiangfei@zs-hospital.sh.cn

Junbo Ge*: jbge@zs-hospital.sh.cn 021-64041990;

Xiangdong Yang*: yangxiangdong_zs@163.com 021-64041990

**This PDF file includes:**

Supplementary Materials and Methods

Figures. S1 to S18

Tables S1 to S7

Captions for Movies S1

Captions for Data S1 to S5

**Other Supplementary Materials for this manuscript include the following:**

Movies S1

Data S1 to S4 [LC-MS/MS_results_mouse_heart_collagen_α1,

LC-MS/MS_results_mouse_heart_collagen_α2,

LC-MS/MS_results_reconstructed_rat_collagen_α1,

LC-MS/MS_results_reconstructed_rat_collagen_α2,

Directly detection of histaminylation in rat-tail type I collagen,

Original and uncropped films of Western blots]

Materials and Methods

**Experimental Design**

Hdc^-/-^ mice, which are deficient in endogenous histamine, were employed alongside wild-type controls to establish and compare an AMI model. To confirm the presence of collagen histaminylation, cardiac collagen was first extracted from post-AMI cardiac tissue by pepsin digestion. Following SDS-PAGE separation, Type I collagen bands were excised for LC-MS/MS analysis. Collagen ultrastructure was then examined by SEM, TGM2-mediated collagen crosslinking levels were quantified using an AQMC assay, and the mechanical properties of the infarcted matrix were assessed with an Optics11 Piuma nanointender. Through these combined approaches, we confirmed that Type I collagen undergoes histaminylation after AMI and observed significant alterations in both collagen ultrastructure and matrix mechanical characteristics in Hdc^-/-^ mice. To delineate the populations of Hdc^+^ cells, we analyzed the publicly available scRNA-seq dataset GSE163129 and established an AMI model with Hdc-GFP transgenic mice. In post-AMI cardiac tissue, histamine production is primarily attributed to neutrophils and a macrophage subpopulation characterized by high HDC expression, with the bulk of release occurring during the early post-AMI period (days 1-3). Subsequently, we engineered collagen matrices in vitro with and without histaminylation and employed LC-MS/MS, pepsin digestion assays, nanointender for mechanical characterization, and SEM to assess ultrastructure. These analyses demonstrated that histaminylation of collagen significantly modulates matrix stiffness, viscoelascity, degradability, and ultrastructural organization. To recapitulate the unique mechanical milieu of the myocardium, we utilized a uniaxial cell-stretching apparatus and seeded primary NMCFs onto natural self-assembled collagen matrices (NC), unmodified-collagen (UC), and histaminylated-collagen (HC) substrates. FMT was induced by combined TGF-β stimulation and cyclic stretch. Subsequent analyses by transcriptome sequencing, immunofluorescence, and Western blotting revealed that histaminylated collagen (HC) markedly attenuated FMT compared with NC and UC conditions. We integrated an aggregated snRNA-seq dataset of AMI patients and healthy donors with our local bulk RNA-seq of post-AMI cardiac tissue between WT and Hdc^-/-^ mice to perform correlation analyses of mechanotransduction signaling between FMT. Our findings indicate that Piezo1-mediated mechanotransduction is likely closely associated with the collagen-binding integrin Itgb1, FMT, and collagen secretion. NMCFs seeded on different collagen substrates were transfected with Piezo1-targeting siRNA or treated with the Piezo1 agonist Yoda1 to verify that histaminylated collagen attenuates FMT by inhibiting Piezo1-mediated mechanotransductive signaling. Finally, to counteract the ECM stiffening and loss of viscoelasticity caused by deficient collagen histaminylation, we locally administered an injectable dopamine (DA)-crosslinked hyaluronan (HA) hydrogel formulated for histamine delivery in Hdc^-/-^ mice following AMI. Concurrently, we treated a separate cohort with the Piezo1 inhibitor GsMTx4. Cardiac outcome and tissue mechanics were evaluated by Masson’s trichrome staining, transthoracic echocardiography, and measurement of myocardial Young’s modulus. Finally, LC-MS/MS analysis was performed to confirm whether collagen histaminylation was restored after histamine supplementation.

**AMI model**

The AMI mouse model was generated via permanent ligation of the left anterior descending (LAD) artery, following established protocols^1^. Eight weeks old mice were anesthetized with intraperitoneal Avertin (SigmaAldrich), intubated using a 22G catheter, and ventilated with a small-animal respirator. A left thoracotomy was performed between the third and fourth ribs to expose the heart. LAD ligation was performed 1.5-2 mm below the left auricle using an 6-0 nylon suture. Successful occlusion was indicated by myocardial blanching. Sham-operated controls underwent the same procedure without ligation, and all chest incisions were closed post-surgery. For Piezo1 inhibition, GsMTx4 (MCE) treatment was administered 3 days prior to AMI induction via intraperitoneal injection at a dose of 1 mg/kg.

**Mouse echocardiography**

On 2 days, 1 week, and 3 weeks following AMI induction, mice from each group were randomly selected, anesthetized with an isoflurane/oxygen mixture, and placed supine for transthoracic echocardiography. A veterinary cardiologist blinded to group assignments acquired images with a high-frequency ultrasound system (VisualSonics VeVo 2100 Imaging System, Toronto, Canada) using a 30 MHz probe. Two-dimensional long-axis views were obtained at the level of maximal left ventricular diameter. Left ventricular ejection fraction (LVEF) was then calculated as:


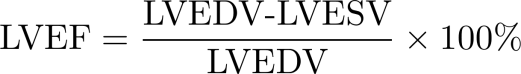
(1-1)


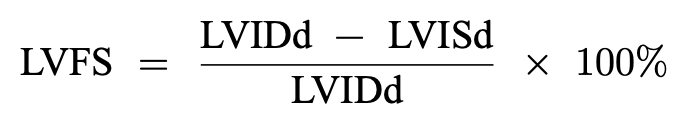
(1-2)

**HPLC-MS/MS**

To quantify the histamine, dopamine, and 5-HT, on 3 days after AMI induction, cardiac tissues below the ligation site and blood samples collected from the orbital venous plexus were obtained from mice. Serum was separated by allowing the samples to stand, followed by centrifugation. The concentrations of three monoamines were determined using HPLC-LC/MS. For tissue samples: Precisely weigh all cardiac tissue samples and transfer them into 2 mL brown centrifuge tubes. Add 0.5 mL of pre-cooled physiological saline, and homogenize at 4 °C for 120 s at 60 Hz, repeating three times. Subsequently, perform ultrasonic extraction in a 4 °C water bath, protected from light, for 30 min. Centrifuge at 13,000 r/min for 20 min at 4 °C, then transfer 0.1 mL of the supernatant to a new 2 mL tube. Add 0.1 mL of LC-MS-grade acetonitrile, vortex for 1 min, and sonicate for 20 min at 4 °C. After another centrifugation (13,000 r/min, 20 min, 4 °C), dilute an appropriate amount of the supernatant 2.5-fold with mobile phase, mix thoroughly, filter through a 0.22 μm membrane, and analyze by LC-MS.For serum samples: Accurately pipette 0.1 mL of mixed serum sample into a 2 mL brown centrifuge tube, add 0.1 mL of LC-MS-grade acetonitrile, vortex for 1 min, and sonicate for 20 min at 4 °C. Centrifuge at 13,000 r/min for 20 min at 4 °C, dilute an appropriate amount of the supernatant 5-fold with mobile phase, mix thoroughly, filter through a 0.22 μm membrane, and subject to LC-MS analysis. Instruments and equipment used: Ultra-high performance liquid chromatography (UHPLC): Waters UPLC; Tandem quadrupole mass spectrometer: Waters XEVO-TQD MS/MS; Chromatographic column: Waters ACQUITY UPLC HSS T3 (2.1 × 100 mm, 1.8 μm). Chromatographic separation conditions:Column temperature: 30 °C; Flow rate: 0.3 mL/min; Autosampler temperature: 10 °C; Mobile phase composition: A: 0.1% formic acid in water, B: methanol; Elution mode: isocratic elution. MS/MS operating conditions: Curtain gas (CUR): 35 psi; Collision gas (CAD): 7 psi; IonSpray voltage: +5500 V; Ionization mode: ESI^+^ (positive electrospray ionization mode); Ion source temperature: 450 °C; Ion source gas 1 (GS1): 40 psi; Ion source gas 2 (GS2): 50 psi. Activated charcoal-purified FBS was used as a blank matrix. A series of mixed standard solutions at different concentrations were prepared in advance and processed following the same sample preparation procedure as the actual samples. The resulting solutions were analyzed to construct a matrix-based calibration curve, which was then used for quantitative determination of monoamines. The processed sample solutions were analyzed using the LC-MS/MS system. The target peak retention times were confirmed based on quality control experiments, and the corresponding peak area data were recorded. The concentrations of the analytes in the actual samples were then calculated according to the internal standard calibration curve and the sample dilution factor. The detailed results are provided in the attached sample data.

**Absolute quantification of matrix-specific crosslinking (AQMC) method**

For the extraction of ECM from post-AMI cardiac tissue, we adopted and modified previously described methods^2^. Briefly, the infarcted myocardium distal to the ligation site was embedded in optimal cutting temperature (OCT) compound and rapidly frozen in liquid nitrogen. The frozen samples were sectioned transversely into 100 µm slices using a cryostat and subsequently incubated overnight on a horizontal shaker at room temperature in a solution containing 2% Triton X-100 and 20 mM EDTA prepared in double-distilled water. The decellularized ECM was then rinsed five times with PBS to remove residual reagents. For the in vitro collagen matrix, approximately 1 mg of collagen was weighed and lyophilized. The enrichment of γ-Glu-ε-Lys was performed following a previously reported stepwise enzymatic digestion protocol^3^ with minor modifications. Briefly, approximately 1 mg of sample was treated sequentially every 24 h with the following enzyme preparations: (1) 2 mg/ml collagenase I (Worthington) in 1 ml Tris-HCl buffer (pH 8); (2) 0.8 U pronase (Roche); (3) 0.8 U pronase and 0.5 mg papain (Sigma); (4) 0.8 U aminopeptidase M (Sigma), 1 U prolidase (Sigma), and 10 mM Mg^2+^/Mn^2+^ prepared from MgCl_2_ and MnSO_4_ (Macklin); (5) 0.8 U aminopeptidase M; and (6) 0.65 U carboxypeptidase (Sigma). To prevent bacterial growth, thymol (1 mg/ml, Macklin) was added to each sample. The total reaction volume was maintained at about 1 ml. Samples were incubated in a shaking incubator at 37 °C, and after each step, the enzymes were inactivated by vortexing and ultrasonication at 100 °C for 10 min. Following enzymatic digestion, residual enzymes were removed by cold methanol precipitation (-80 °C, 1 h) and centrifugation (13,000 g, 15 min). The supernatant, containing collagen crosslinks, was collected and lyophilized, then dissolved in lysate (8 M urea, 0.1 M Tris-HCl pH 8.5). The γ-Glu-ε-Lys standard was obtained from Bioleaper (Shanghai, China) (Figure. S17). Analysis conducted by HPLC-MS/MS using the same methods as described above. The extent of TGM2-mediated crosslinking was quantified as the molar ratio between γ-Glu-ε-Lys and type I collagen within each sample.

**Collagen degradation test**

Degradation of in vitro constructed collagen matrix was induced by 0.08% collagenase type I (Worthington). In brief, the collagen mixture was added to 96-well culture plates (Corning) and gelled at 37 °C. The turbidity at 400 nm^4^ was measured using VarioskanLux (thermos scientific) and recorded as the baseline value. Excess collagenase was then added to each well to fully cover the gel surface, followed by incubation on a shaker at 37 °C. Turbidity was measured every 30 min, and the percentage decrease in turbidity was calculated as the degradation ratio.

**ELISA**

The concentrations of IL-1β and IL-6 were measured using enzyme-linked immunosorbent assay (ELISA) kits (BOSTER). All ELISA procedures were performed according to the manufacturer’s instructions, and absorbance was recorded using VarioskanLux (Thermo Scientific).

**Flow cytometric analysis**

Blood, spleen, bone marrow, and cardiac muscle were collected from Hdc-GFP, Hdc^-/-^ and WT mice on day 3 and day 7 following AMI. Peripheral blood samples were subjected to red blood cell lysis, spleens were mechanically dissociated, and bone marrow cells were flushed from the femurs with PBS (Sangon Biotech). Cardiac muscle specimens were digested at 37 °C for 15 minutes with type I collagenase (3 mg/mL) and neutral protease (4 mg/mL) (Worthington), then passed through a 40 µm cell strainer. For flow cytometric labeling, 1 µg of fluorophore-conjugated antibody was added per 10^6^ cells and incubated at 4 °C for 60 minutes. Two antibody panels, as detailed in the Supplemental Information, were used to profile immune cell populations (Supplementary Table S4).

**Neonatal mouse CFs and cardiomyocytes isolation**

Neonatal Balb/c mice (1-3 days old) were euthanized, and hearts were immediately excised and rinsed in cold Hank’s balanced salt solution (HBSS) to remove blood. Ventricles were minced and incubated in 0.01 mg/mL trypsin (Gibco) in HBSS at 4 °C overnight. The following day, pre-digested tissue was subjected to 1500 U/mL collagenase type I (Worthington) for 17 min at 37 °C with gentle agitation. The resulting cell suspension was triturated, filtered through a 70 μm strainer, centrifuged, and resuspended in high-glucose DMEM (Gibco) containing 10% fetal bovine serum (Biological Industries). Cells were plated in 10 cm dishes and incubated at 37 °C with 5% CO_2_ for 1.5 h; adherent cells were collected as cardiac fibroblasts (NMCFs), The suspended cells were collected as cardiomyocytes (NMCMs).

**Immunofluorescence**

Mouse heart tissues were fixed in 4% paraformaldehyde and embedded in optimal cutting temperature (OCT) compound. Following freezing, tissues were cryosectioned into 7 μm slices at −25 °C. For NMCFs culture, 10 μL of the collagen mixture was added to each well of a 15-well glass-bottom plate (ibidi), or 400 μL for the 6-well soft-bottom plate (Flex Cell). Cells were fixed with 4% paraformaldehyde and permeabilized using 0.2% Triton X-100. Primary antibodies were incubated overnight at 4 °C, followed by secondary antibody incubation at 37 °C for 1 hour after PBS washes. Nuclei were counterstained with DAPI. For histological analysis, mouse heart samples were fixed in 4% formalin, paraffin embedded, and sectioned for H&E and Masson’s trichrome staining. A list of antibodies is provided in Supplementary Table S5.

**RNA Extraction and Quantitative RT-PCR**

Total RNA was isolated from cells enzymatically released from the collagen matrix using type I collagenase, followed by extraction with TRIzol reagent (Takara). cDNA synthesis was performed using the PrimeScript™ RT reagent kit (Takara), and qPCR was carried out with the TB Green™ Premix Ex Taq™ kit (Takara) on the QuantStudio 5 system (ABI). Gene expression levels were normalized to Gapdh, and results were calculated independently for each target gene. Primer sequences, designed based on GeneBank entries from PubMed and synthesized by Huagene Biotech, are listed in Supplementary Table S6.

**Western blot analysis**

Fibroblasts were released from the collagen matrix using type I collagenase digestion, then washed, centrifuged, and lysed in ice-cold buffer supplemented with protease and phosphatase inhibitor cocktails. Protein concentrations were quantified using the BCA Protein Assay Kit. Equal amounts of total protein were separated by 10% SDS-PAGE and transferred onto PVDF membranes. Membranes were incubated with primary antibodies overnight at 4 °C, followed by HRP-conjugated secondary antibodies at 37 °C. Signal detection was performed using a ChemiScope chemiluminescence imaging system. Detailed information on the primary antibodies used is provided in Supplementary Table S5.

**scRNA-seq data analysis**

The primary analysis of single-cell RNA-sequencing (scRNA-seq) data was conducted using the R package Seurat (version 4.4.0)^5^. Cells with fewer than 200 detected genes or genes expressed in fewer than three cells were excluded. Additionally, cells with more than 5% mitochondrial gene content or over 50% ribosomal gene expression were filtered out. The R package Scater (version 1.30.1) and its "isOutlier" function were applied to identify outliers in gene counts. Potential doublets were removed using scDblFinder (version 1.14.0) with a threshold of 0.075%. Data integration across samples was performed using Harmony (version 1.2.0), and decontamination was addressed with decontX (version 1.0.0). Cell clustering was performed via the "FindClusters" function in Seurat, and marker genes were identified using "FindAllMarkers". Cell type annotation was carried out with SingleR (version 2.2.0), with manual verification to ensure accuracy. Trajectory inference was performed using Monocle3 (version 1.3.4), and gene expression dynamics along pseudotime as well as gene co-expression modules were analyzed using the "track_genes" and "find_gene_modules" functions of Monocle3. Data analysis and visualization were completed with SCP (version 0.5.6).

**RNA-seq and data processing**

Total RNA was isolated from WT and Hdc^-/-^ mouse hearts on 1 and 7 days post-surgery using TRIzol reagent (Takara) for RNA-seq analysis. Sequencing read quality was initially assessed with FastQC (version 0.11.9) following preliminary quality control (version 0.20.1). Gene expression was quantified by aligning reads to the reference genome using HISAT2 (version 2.1.0) and counting with HTSeq-count (version 0.11.2). Expression values were normalized to FPKM to account for gene length and sequencing depth. The resulting count matrix was analyzed in R using DESeq2 (version 1.22.2) for normalization and dispersion estimation. Differentially expressed genes (DEGs) were defined by p-values < 0.05 and log2 fold change > 0.25. KEGG pathway enrichment analysis was performed with clusterProfiler (version 4.13.0), and results were visualized using ggplot2 (version 3.4.4) and pheatmap (version 1.0.12).

**Histaminylated sites motif analysis**

After database searching, the modified sites were identified by examining the “Modifications in Master Proteins” column in the result table, and the positions of histaminylated glutamine (Q) residues were recorded. Using the reference protein sequence, we extracted 21-amino-acid peptides centered on each modified Q (±10 amino acids). Conservation analysis of these peptides was performed using the BLASTP tool (https://blast.ncbi.nlm.nih.gov/Blast.cgi?PAGE=Proteins). For motif analysis, the extracted peptide sequences were analyzed using the MEME Suite^6^. To generate a negative dataset, all Q residues from type I collagen together with their ±10 flanking amino acids were extracted using a Python script, and CD-HIT was applied to compare these peptides with the positive histaminylated peptides. Peptides with <40% sequence similarity to any positive peptide were defined as negative sites. The resulting positive and negative datasets were then subjected to comparative motif analysis using the TwoSampleLogo suite^7^.

**Mathematics model**

The detailed parameters of FMPCL had been reported in the previous study^8^. The model was calculated based on following formulas:


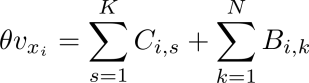
 (1)


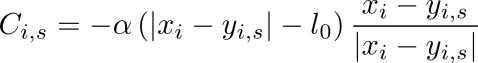
(1-2)


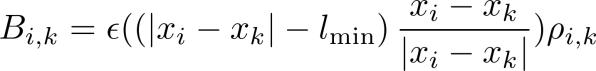
(1-3)


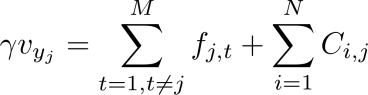
(2)


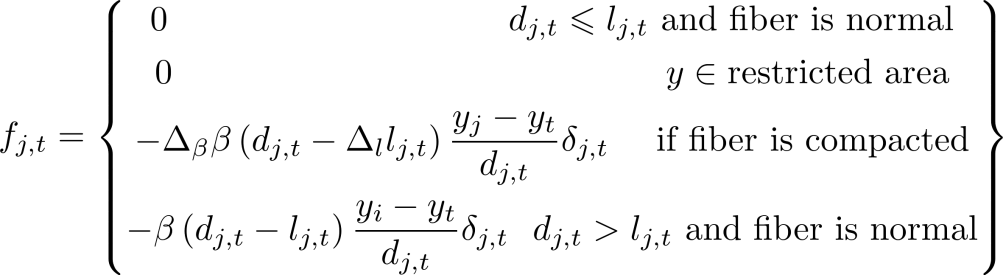
(2-1)


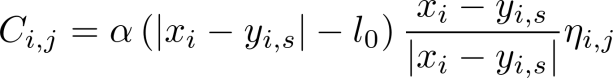
(2-2)


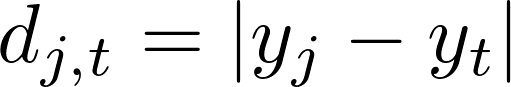
(2-3)

Fibroblast activation is implemented phenomenologically: if any binding site in a quiescent cell experiences ≥0.8 × (myofibroblast peak contraction) for ≥25 min, the cell converts to a myofibroblast within the next hour. The collagen matrix was constructed using scipy.spatial.Delaunay, and all computations were performed using PyTorch (version 2.8.6) with CUDA acceleration (version 12.6).

**Scanning Electron Microscope and Transmission Electron Microscope**

Tissue samples were trimmed to ≤1 mm^3^ to minimize mechanical damage and fixed in EM fixative at 4 °C for 2–4 hours. Collagen matrices seeded with cells were similarly cut and fixed. All samples were washed three times with 0.1 M PBS (pH 7.4, 15 min each), post-fixed in 1% osmium tetroxide (RT, 2 h), and rewashed in PBS. Dehydration was performed using a graded ethanol series (50%–100%, 15 min each). Samples were infiltrated overnight with a 1:1 mixture of acetone and Epon 812, followed by pure resin infiltration and embedding at 60 °C for 48 h. Ultrathin sections (60–80 nm) were cut with an ultramicrotome, stained with 2% uranyl acetate and lead citrate (15 min each), and air-dried. Transmission electron microscopy (TEM, FEI Tecnai G2 20 TWIN) was used to examine ultrastructure. Where applicable, scanning electron microscopy (SEM, HITACHI SU8100) was employed in parallel to visualize surface morphology.

**siRNA transfection**

Piezo1 and Itgb1 specific siRNA and negative control siRNA sequences were designed and synthesized by AnzhenBio Co., Ltd. Three target sequences of Piezo1 siRNA used in mice NMCFs were as follows: Piezo1 siRNA (1) sense strand (SS): GCGGACTTAAACTGCAATA, antisense strand (AS): UUAGCUUCUGGCUCUUCCUTT; Piezo1 siRNA (2) SS:GUGCCAAACAGGAGAAGUATT, AS: UACUUCUCCUGUUUGGCACTT; Piezo1 siRNA (3) SS: GCAAGACUGUCCUGGGAAATT, AS: UUUCCCAGGACAGUCUUGCTT; si-RNA NC SS: UUCUCCGAACGUGUCACGUTT, AS: ACGUGACACGUUCGGAGAATT. Three target sequences of Itgb1 siRNA used in mice NMCFs were as follows: Itgb1 siRNA (1) SS: GCACGAUGUGAUGAUUUAGAATT, AS: UUCUAAAUCAUCACAUCGUGCTT; Itgb1 siRNA (2) SS: GCCAUUACUAUGAUUAUCCUUTT, AS: AAGGAUAAUCAUAGUAAUGGCTT. Itgb1 siRNA (3) SS: CCCGACAUCAUCCCAAUUGUATT, AS: UACAAUUGGGAUGAUGUCGGGTT. Itga2 siRNA knockdown was conducted by Itga2 Mouse Pre-designed siRNA Set A (MCE). siRNA transfection was performed as the instruction of Lipofectamine® RNAiMAX Reagent (Invitrogen, 13778075). At the culmination of a 48-hour period following siRNA transfection, cells were gathered and subjected to analysis via qRT-PCR and western blotting.

**Hydrogel preparation and characterization**

HA-DA hydrogel was synthesized as previously described^9^. Briefly, 1.0 g hyaluronic acid (HA) was dissolved in 100 mL deionized water under nitrogen. EDC-HCl (1.48 g) and NHS (0.9 g) were added and stirred for 20 min. Dopamine (1480 mg) and histamine diphosphate (1000 mg) were then introduced, followed by rapid mixing for 1 min. The pH was adjusted to 5.0-.5, and unreacted compounds were removed via 48 h dialysis (MWCO 8000-14000 Da). Hydrogel morphology was assessed using SEM. For swelling analysis, samples were incubated in PBS at 37 °C, blotted, and weighed. Degradation was evaluated by freeze-drying at -20 °C to determine initial weight (W_0_), followed by PBS incubation at 37 °C. At set time points, samples were reweighed (W_t_), and degradation was calculated as:


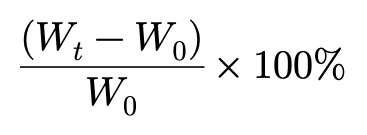
(3-1)

After preparation, The hydrogel (≈80 µL in total) was injected to four peri-infarct locations (two along the anterior wall, one at the lateral wall, and one at the apex) immediately following LAD ligation in mice^2^.

**References**

1. Gao, E. *et al*. A novel and efficient model of coronary artery ligation and myocardial infarction in the mouse. *Circ. Res.* **107**, 1445–1453 (2010).
2. Feng, J. *et al*. Versican promotes cardiomyocyte proliferation and cardiac repair. *Circulation* **149**, 1004–1015 (2024).
3. Lyu, C. *et al*. Advanced glycation end-products as mediators of the aberrant crosslinking of extracellular matrix in scarred liver tissue. *Nat. Biomed. Eng.* **7**, 1437–1454 (2023).
4. Cortes-Medina, M. *et al*. Chondroitin sulfate, dermatan sulfate, and hyaluronic acid differentially modify the biophysical properties of collagen-based hydrogels. *Acta Biomater.* **174**, 116–126 (2024).
5. Hao, Y. *et al*. Integrated analysis of multimodal single-cell data. *Cell* **184**, 3573–3587 (2021).
6. Bailey, T. L., Johnson, J., Grant, C. E. & Noble, W. S. The MEME suite. *Nucleic Acids Res.* **43**, W39–W49 (2015).
7. Crooks, G. E., Hon, G., Chandonia, J. M. & Brenner, S. E. WebLogo: a sequence logo generator. *Genome Res.* **14**, 1188–1190 (2004).
8. Liu, L. *et al*. Matrix-transmitted paratensile signaling enables myofibroblast-fibroblast cross talk in fibrosis expansion. *Proc. Natl. Acad. Sci. U. S. A.* **117**, 10832–10838 (2020).
9. Wang, Y. *et al*. Versatile dopamine-functionalized hyaluronic acid-recombinant human collagen hydrogel promoting diabetic wound healing via inflammation control and vascularization tissue regeneration. *Bioact. Mater.* **35**, 330–345 (2024).


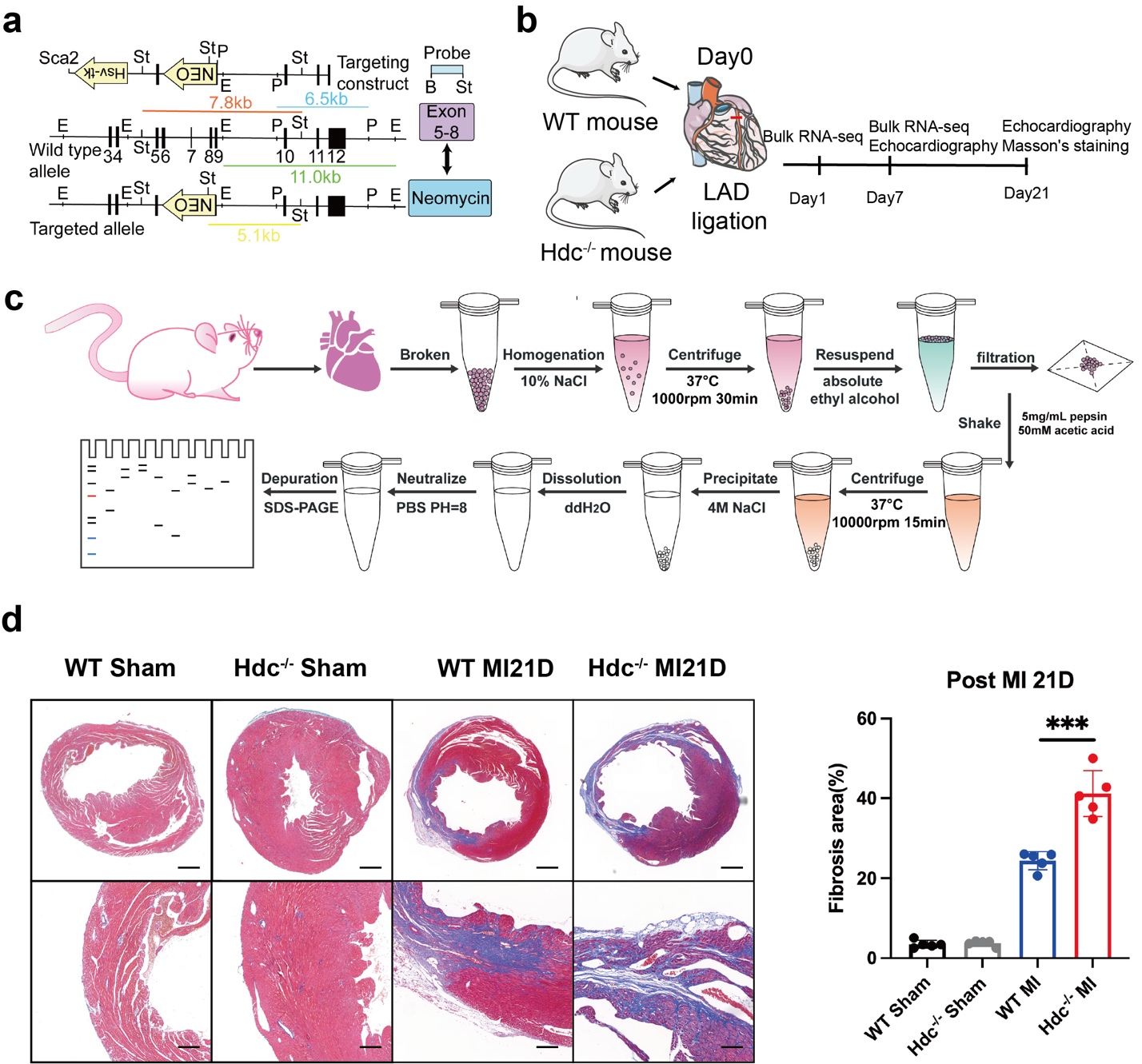


**Figure. S1. Endogenous histamine deficiency in Hdc^-/-^ mice resulted in significantly exacerbated fibrosis and impaired cardiac function following AMI.** **a** The schematic of the Hdc^-/-^ mice construction. **b** The schematic of AMI model and examination. **c** The schematic of collagen extraction and purification from mouse heart post-AMI. **d** Representative image of Masson staining and quantitative analysis of cardiac fibrosis area (n = 5; scale bar: top, 100 μm; bottom 25μm). For all experiments, error bars represent the mean ± SD. *P < 0.05, **P < 0.01, ***P < 0.001, ****P < 0.0001


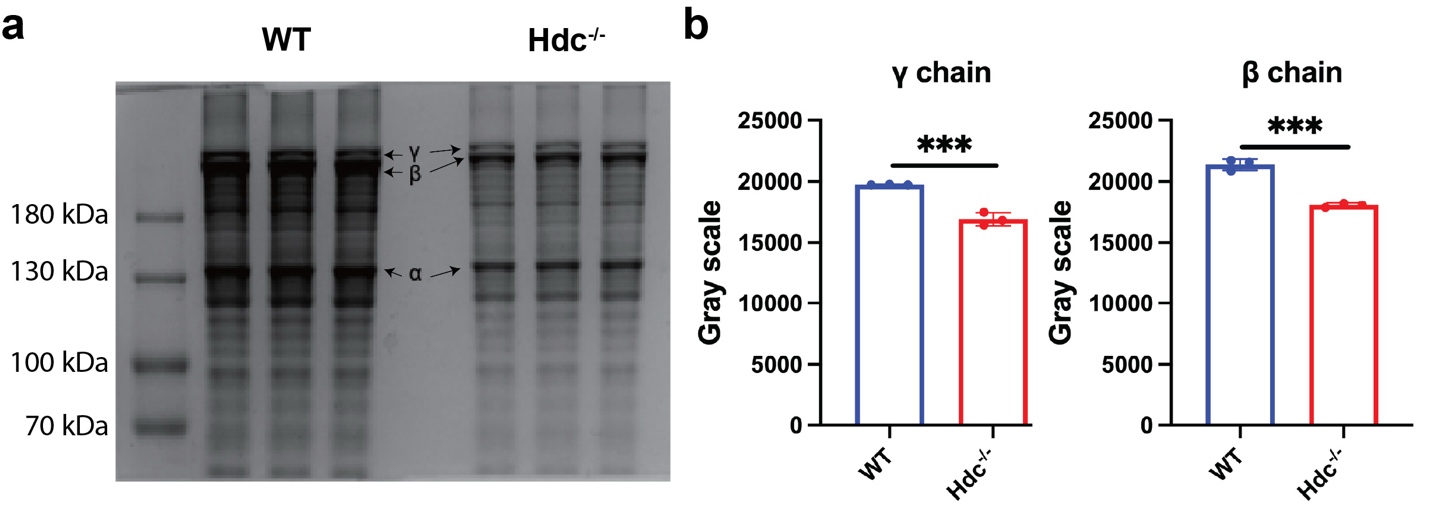


**Figure. S2. Endogenous histamine deficiency in Hdc^-/-^ mice significantly reduced the extractability of cardiac collagen. a** Representative image of SDS-page and Coomassie Brilliant Blue staining of extracted collagen chains from post-AMI cardiac tissue. **b** Quantitative analysis of gray scale of Coomassie Brilliant Blue staining (n = 3). For all experiments, error bars represent the mean ± SD. *P < 0.05, **P < 0.01, ***P < 0.001, ****P < 0.0001.


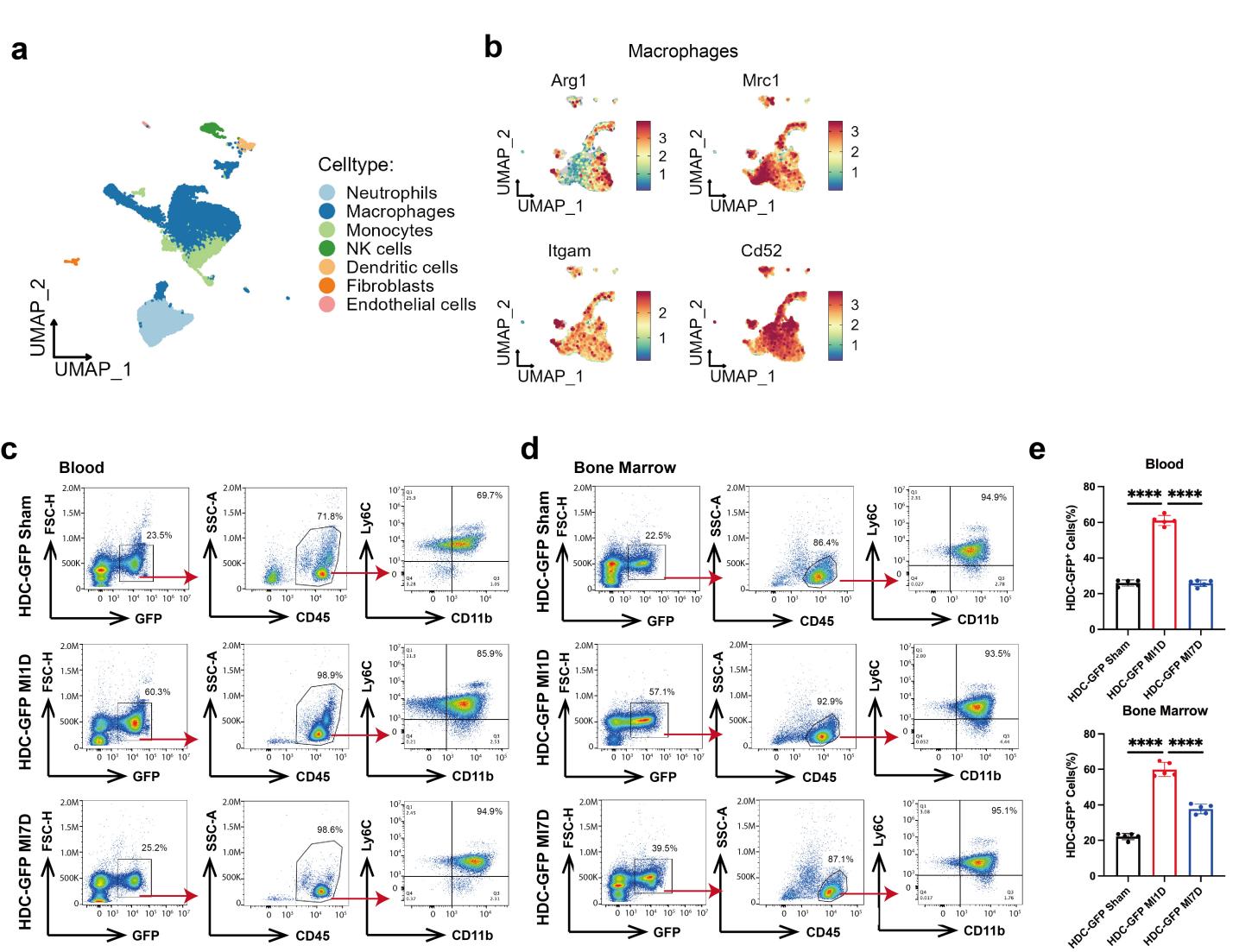


**Figure. S3.The local histamine following AMI is primarily derived from early-infiltrating Hdc-expressed neutrophils and macrophages with high. a** Clustering and cell type annotation of CD45^+^ immune cells. **b** Multiple macrophage marker genes are highly expressed in post-AMI macrophage populations. **c** Representative flow cytometry images of GFP^+^ cell in peripheral blood from Hdc-GFP mice at Sham and on day 1 and day 7 post-AMI. **d** Representative flow cytometry images of GFP^+^ cell in bone marrow from Hdc-GFP mice at Sham and on day 1 and day 7 post-AMI. **e** Quantitative analysis of flow cytometry of GFP^+^ cell form peripheral blood and bone marrow (n = 5). For all experiments, error bars represent the mean ± SD. ****P < 0.0001.


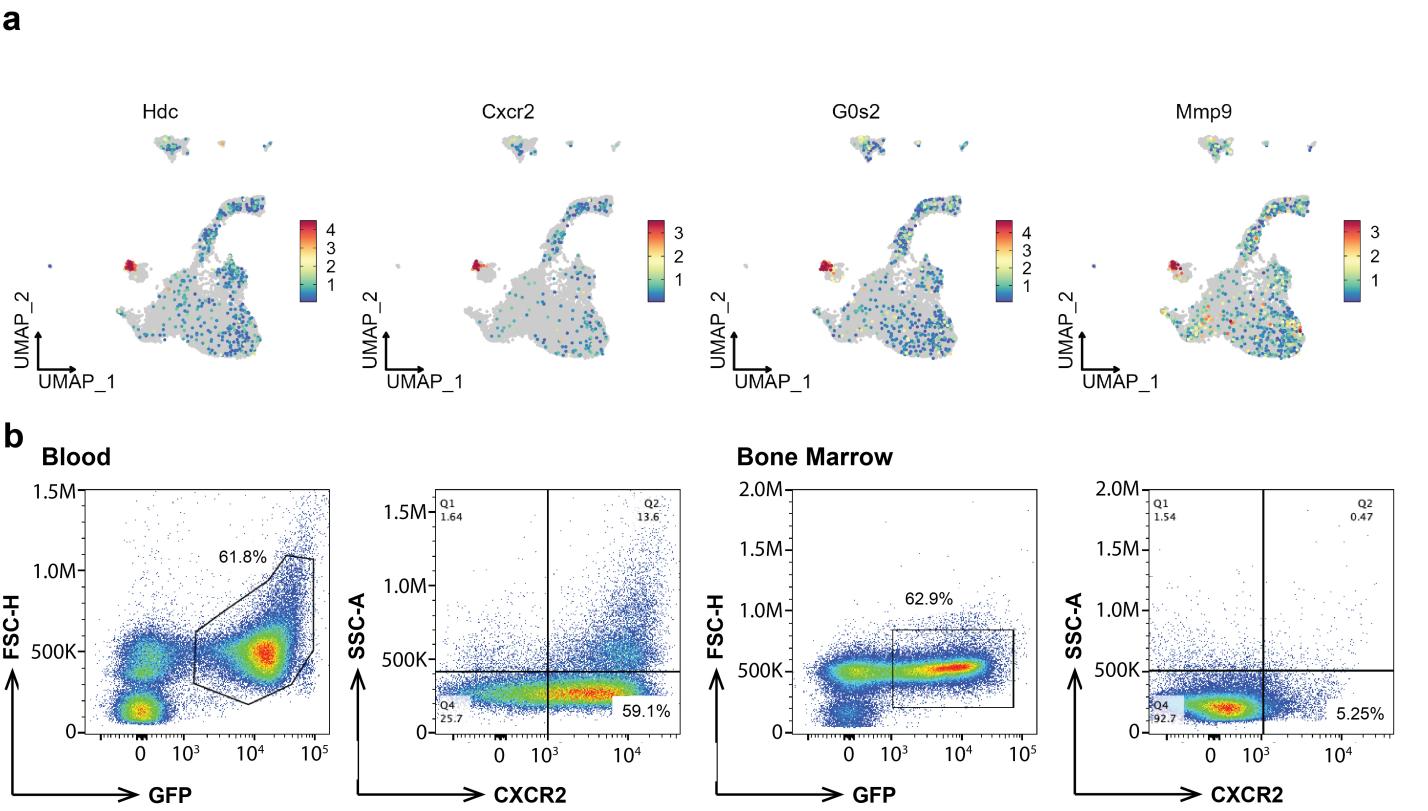


**Figure. S4. Characterization of Hdc^+^ macrophages**

**a** Sc-RNAseq analysis reveals that Hdc^+^ macrophages co-express high levels of Cxcr2, G0s2, and Mmp9. **b** Flow cytometry analysis of peripheral blood and bone marrow demonstrates that a substantial proportion of GFP^+^ cells recruited to the peripheral blood express high levels of CXCR2 (on day3 post-AMI).


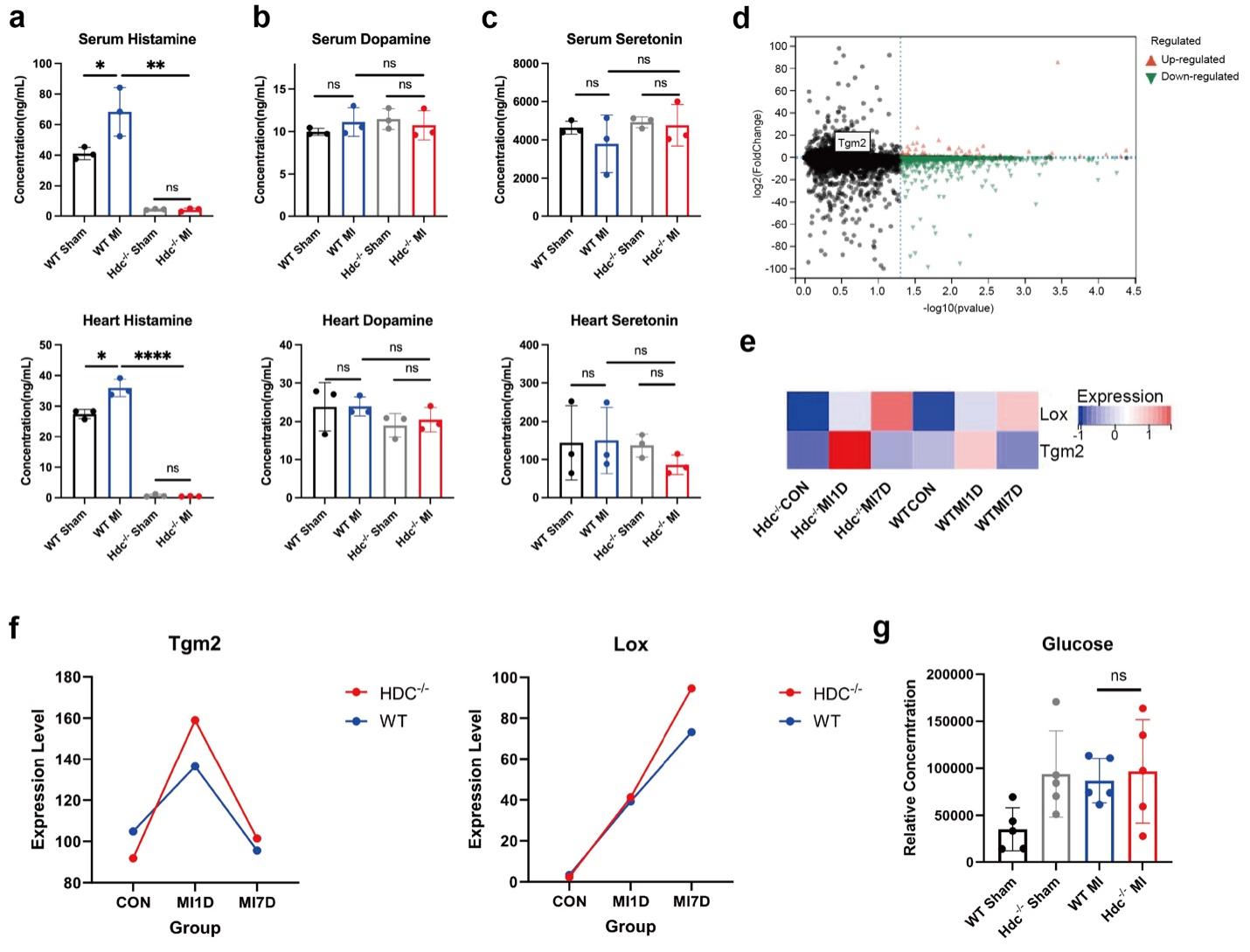


**Figure. S5. The differential histamine levels, rather than other monoamines or forms of collagen crosslinking, are the primary factor influencing the post-infarction outcomes in Hdc^-/-^ mice.**

**a** Quantitative analysis of HPLC-MS/MS test of serum and heart tissue dopamine (DA) on day 7 post-AMI. **b** Quantitative analysis of HPLC-MS/MS test of serum and heart tissue histamine (HA) on day 7 post-AMI. **c** Quantitative analysis of HPLC-MS/MS test of serum and heart tissue serotonin (5HT) on day 7 post-AMI. **d** Volcano plot showed the bulk-RNAseq DEGs of post-AMI heart tissue on day 7 post-AMI between Hdc^-/-^ mice and WT mice labeled with TGM2 which shows no difference. **e** Heatmap of Lox and Tgm2 of post-AMI heart tissue bulk-RNAseq on day 1 and day 7 post-AMI. **f** Line chart showed expression level of Lox and Tgm2 in heart tissue bulk-RNAseq on day 1 and day 7 post-AMI. **g** Quantitative analysis of HPLC-MS/MS test of blood glucose on day 7 post-AMI. For all experiments, error bars represent the mean ± SD. *P < 0.05, **P < 0.01, ***P < 0.001, ****P < 0.0001.


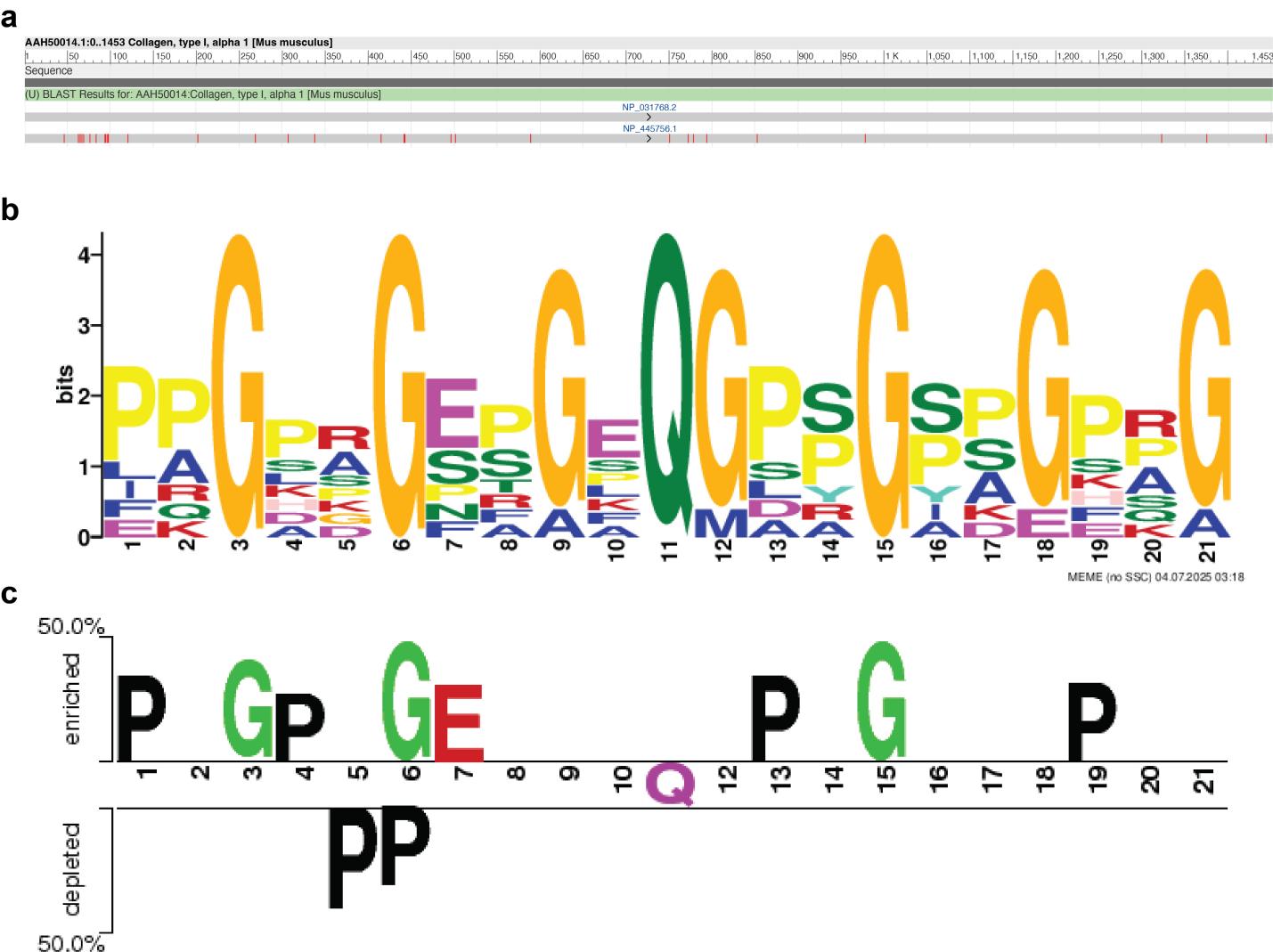


**Figure. S6. Motif analysis of histaminylated Gln residues**

**a** BLASTP result between mouse and rat Type I collagen α1 chain. **b** MEME analysis showed motif of histaminylated Gln residues. **c** Two-sample logo comparison between histaminylated and non-modified Gln residues.


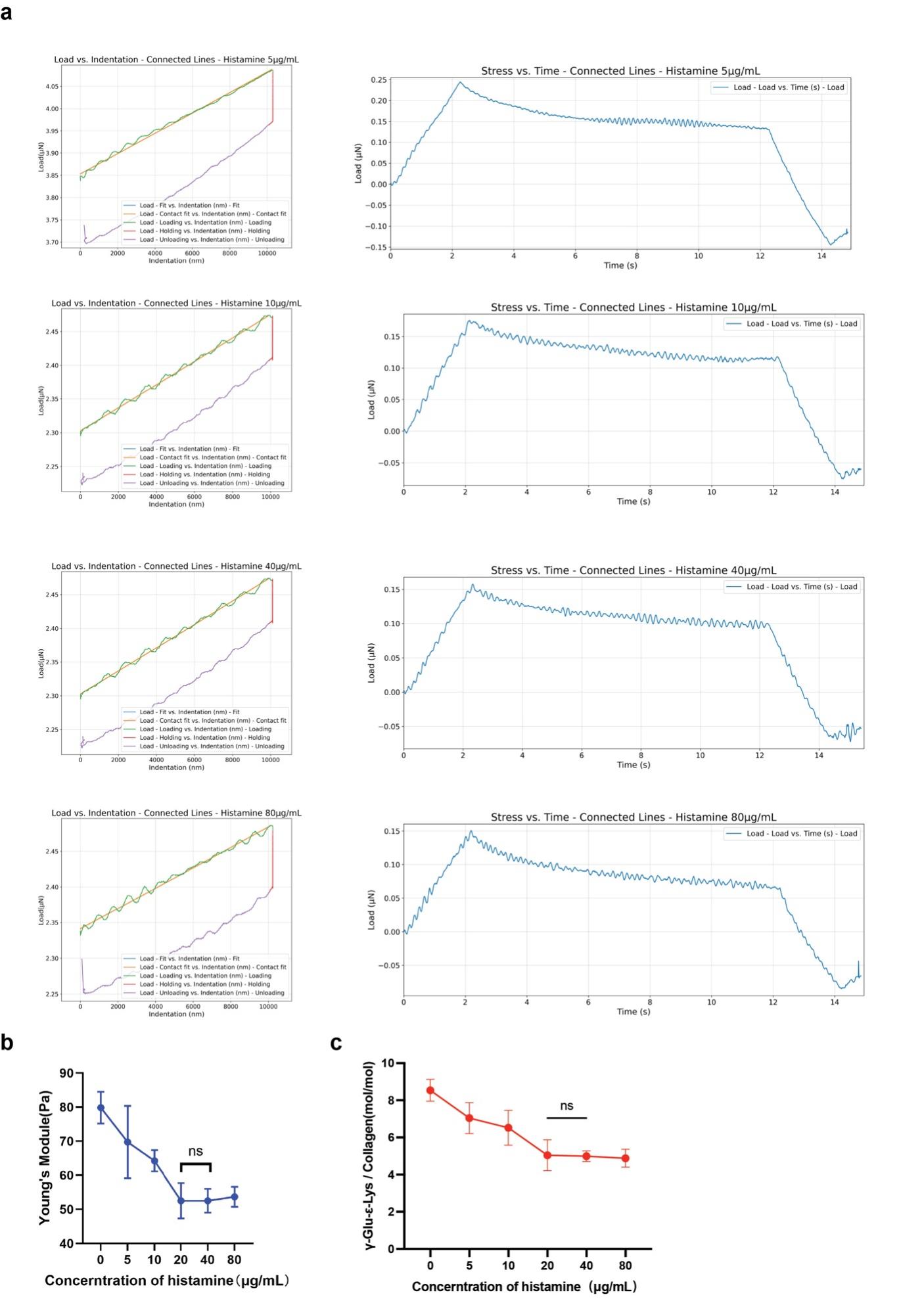


**Figure. S7. Further increasing the histamine concentration beyond the saturation point does not lead to additional changes in the mechanical properties of the collagen matrix.**

**a** Representative plot of load-indentation and stress-relaxation curve of different concentration of histamine. **b** Effect of histamine concentration gradient on the Young’s modulus of collagen matrix measured by nanoindentation (n ≧ 15). **c** Effect of histamine concentration gradient on γ-Glu-ε-Lys as assessed by AQMC assay (n = 5). For all experiments, error bars represent the mean ± SD.


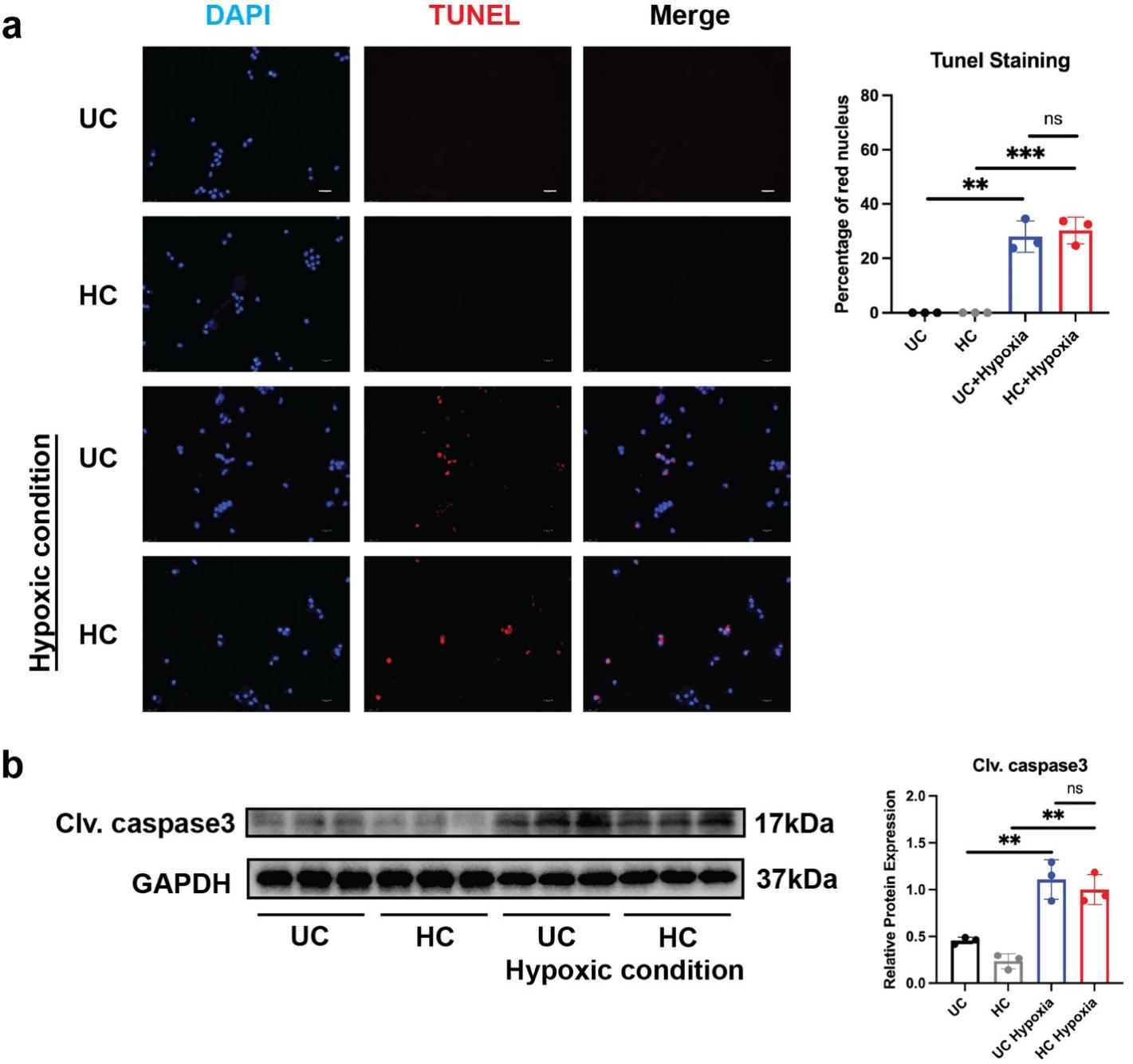


**Figure. S8. HC did not exhibit a significant effect on cardiomyocyte apoptosis.**

1. Representative and quantification analysis of TUNEL staining of NMCMs cultured on HC and UC under hypoxic conditions (n = 3; scale bar, 75 µm). **b.** Representative and quantification analysis of western blot of cleaved Caspase3 (n = 3). For all experiments, error bars represent the mean ± SD. *P < 0.05, **P <0 .01, ***P <0 .001, ****P <0 .0001.


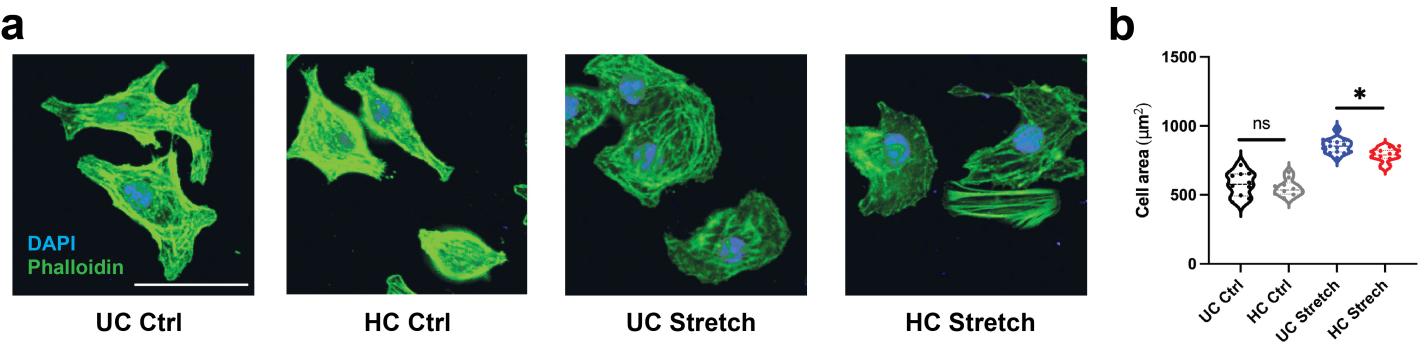


**Figure. S9. Stretch-induced cardiomyocyte hypertrophy exhibited only minor differences between the two collagen matrices.**

**a** Representative image of cell skeleton after cyclic stretching of NMCMs between UC and HC(scale bar, 50μm). **b** Quantification of cell area after cyclic stretching of NMCMs between UC and HC (n ≥ 10)**.** For all experiments, error bars represent the mean ± SD. *P < 0.05, **P < 0.01, ***P < 0.001, ****P < 0.0001.


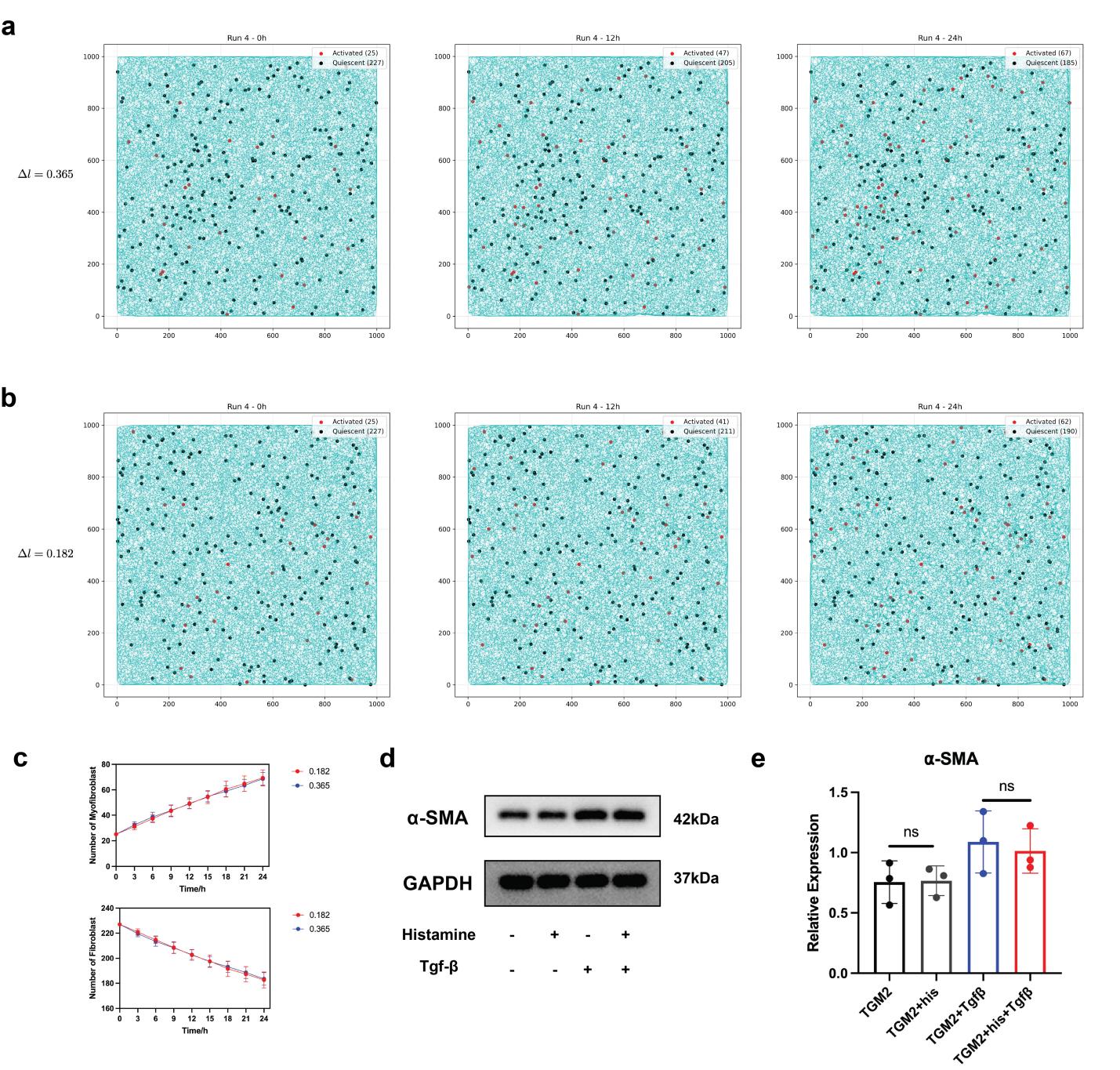


**Figure. S10. No significant difference in FMT was observed between UC and HC under static conditions.**

**a** Simulation of fibroblasts grown on UC at 0, 12, and 24 hours using the FMPCL mathematical model. **b** Simulation of fibroblasts grown on HC at 0, 12, and 24 hours using the FMPCL model. **c** Statistical analysis of simulation runs using different random seeds for each condition (n = 20). **d** Western blot analysis of α-SMA protein levels in fibroblasts cultured on UC and HC with TGF-β stimulation. **e** Quantification analysis of western blot results (n = 3). For all experiments, error bars represent the mean ± SD.


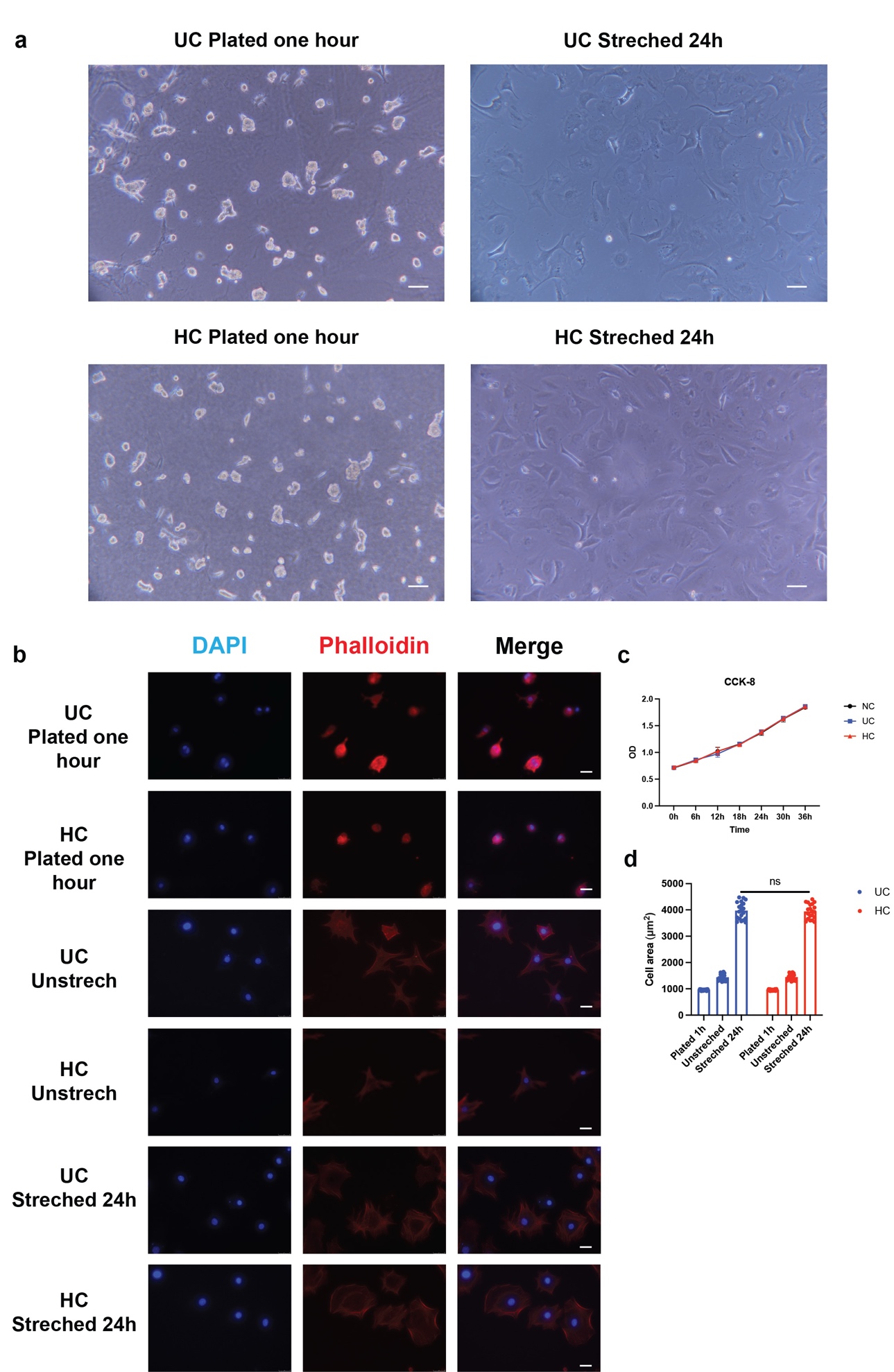


**Figure. S11. Cyclic stretch markedly induces cytoskeletal polarization of NMCFs cultured on collagen matrices.**

**a** Representative bright-field images of NMCFs cultured on UC and HC before and after cyclic stretch (scale bar, 100 μm). **b** Immunofluorescence images of cytoskeletal organization in NMCFs on UC and HC before and after stretch, stained with Phalloidin (scale bar, 75 μm). **c** CCK-8 assay assessing the proliferative capacity of fibroblasts on UC and HC (n = 5). **d** Quantification of cell area in fibroblasts on UC and HC after cyclic stretch (n = 20). For all experiments, error bars represent the mean ± SD. *P < 0.05, **P < 0.01, ***P < 0.001, ****P < 0.0001.


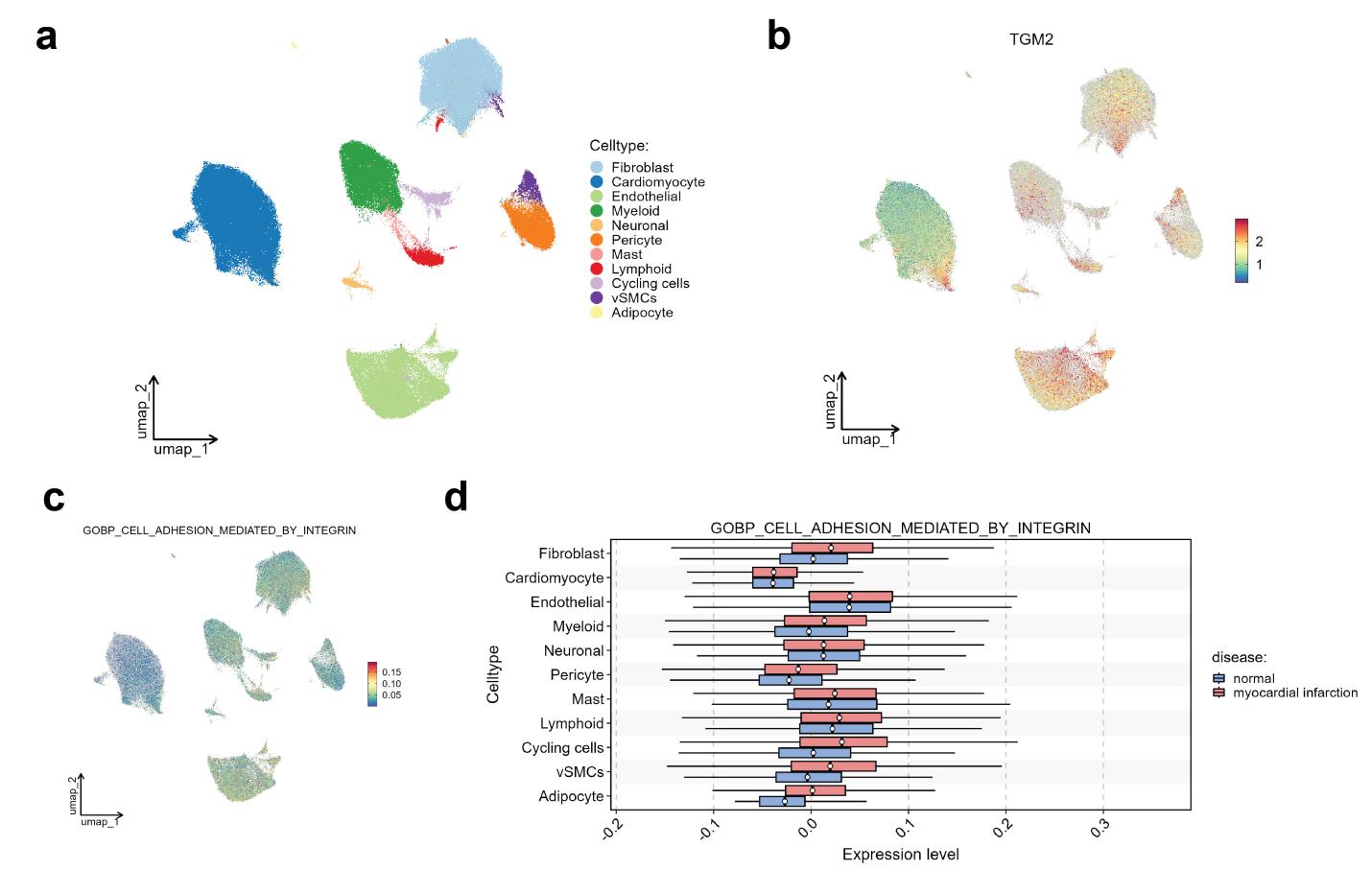


**Figure. S12. Analysis of scRNA-seq datasets from patients with AMI for TGM2 expression and integrin-related FAs formation.** **a** Overall cell clustering and celltype. **b** TGM2 expression levels across cell types. **c** Scores of the GOBP gene set GOBP_CELL_ADHESION_MEDIATED_BY_INTEGRIN in different cell types. **d** Comparison in GOBP_CELL_ADHESION_MEDIATED_BY_INTEGRIN scores between myocardial infarction patients and normal donors across cell types.


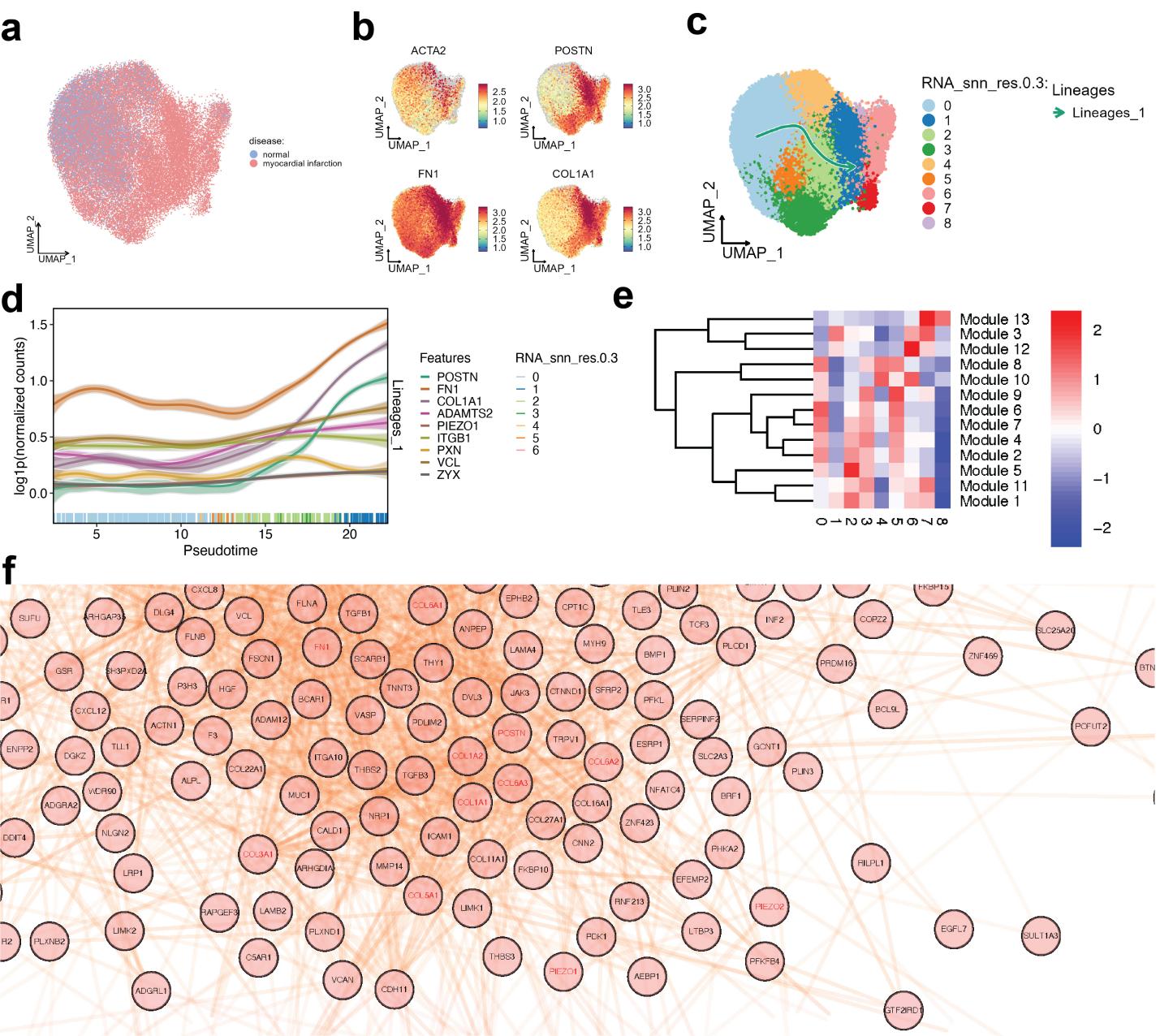


**Figure. S13 scRNA-seq datasets analyses suggest an association between FMT and Piezo1 signaling. a** Dimplot distribution of CFs from healthy donors and MI patients. **b** DimPlot distribution of myofibroblast markers, indicating that Cluster 1 represents the myofibroblast subcluster. **c** Trajectory from healthy quiescent CFs (Cluster 0) to myofibroblasts (Cluster 1). **d** Expression of Itgb1, FAs genes, and myofibroblast-associated markers increased along the pseudotime trajectory. **e** Gene co-expression module analysis indicated that Module 3 was highly expressed in the myofibroblast cluster (Cluster 1). **f** Protein-protein interaction analysis of genes within Module 1 suggested that Piezo1 co-expresses with and may influence various collagen and myofibroblast marker genes.


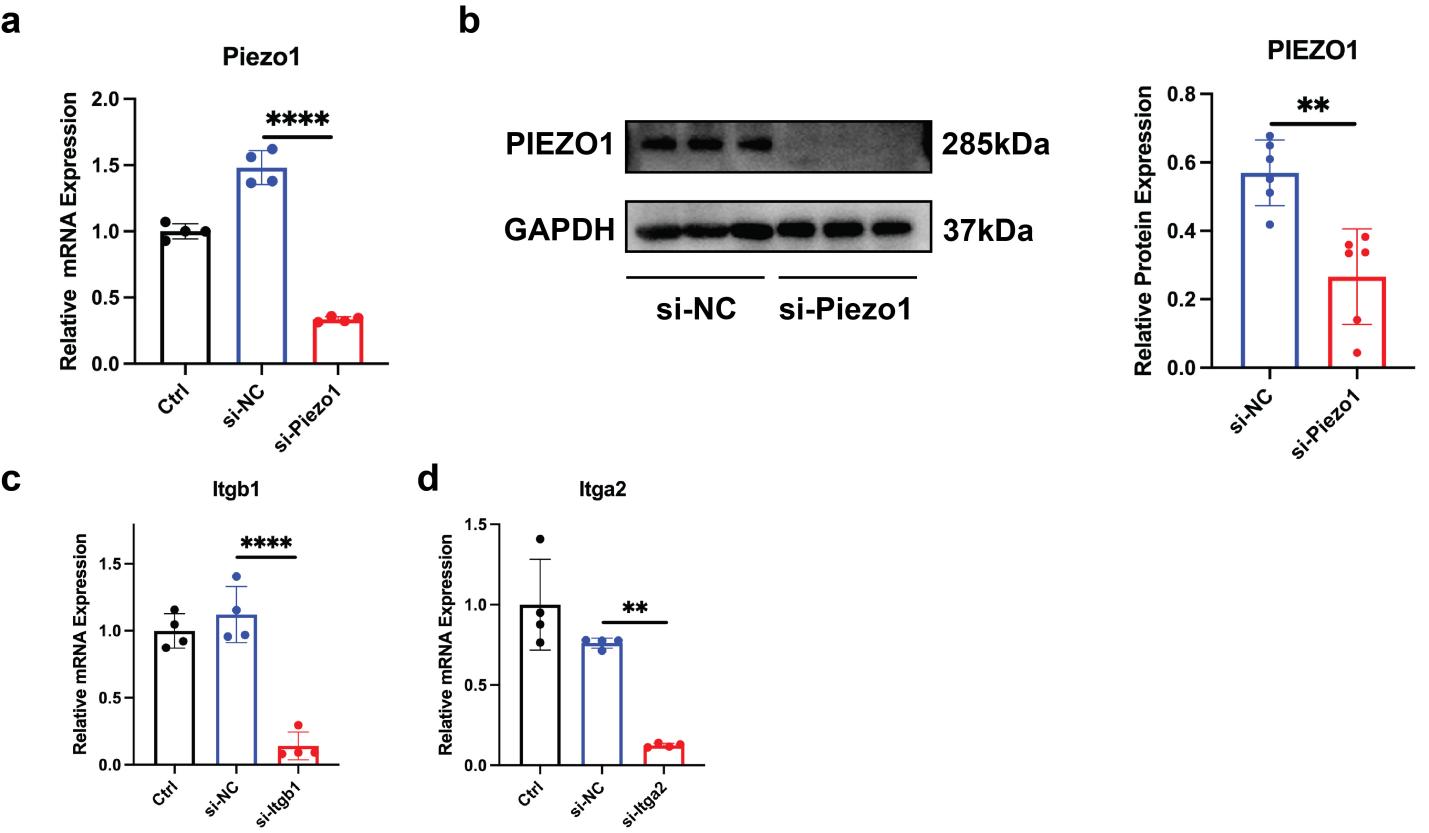


**Figure. S14. Validation of knockdown of Piezo1, Itgb1 and Itga2 in NMCFs using siRNA**

**a** qRT-PCR analysis showing reduced mRNA expression levels of Piezo1 following siRNA-mediated knockdown (n = 4). **b** Representative and quantification of western blot image showed decreased PIEZO1 protein levels after knockdown (n = 6). **c** qRT-PCR analysis showing reduced mRNA expression levels of Itgb1 following siRNA-mediated knockdown (n = 4). **d** qRT-PCR analysis showing reduced mRNA expression levels of Itga2 following siRNA-mediated knockdown (n = 4). For all experiments, error bars represent the mean ± SD. *P < 0.05, **P < 0.01, ***P < 0.001, ****P < 0.0001.


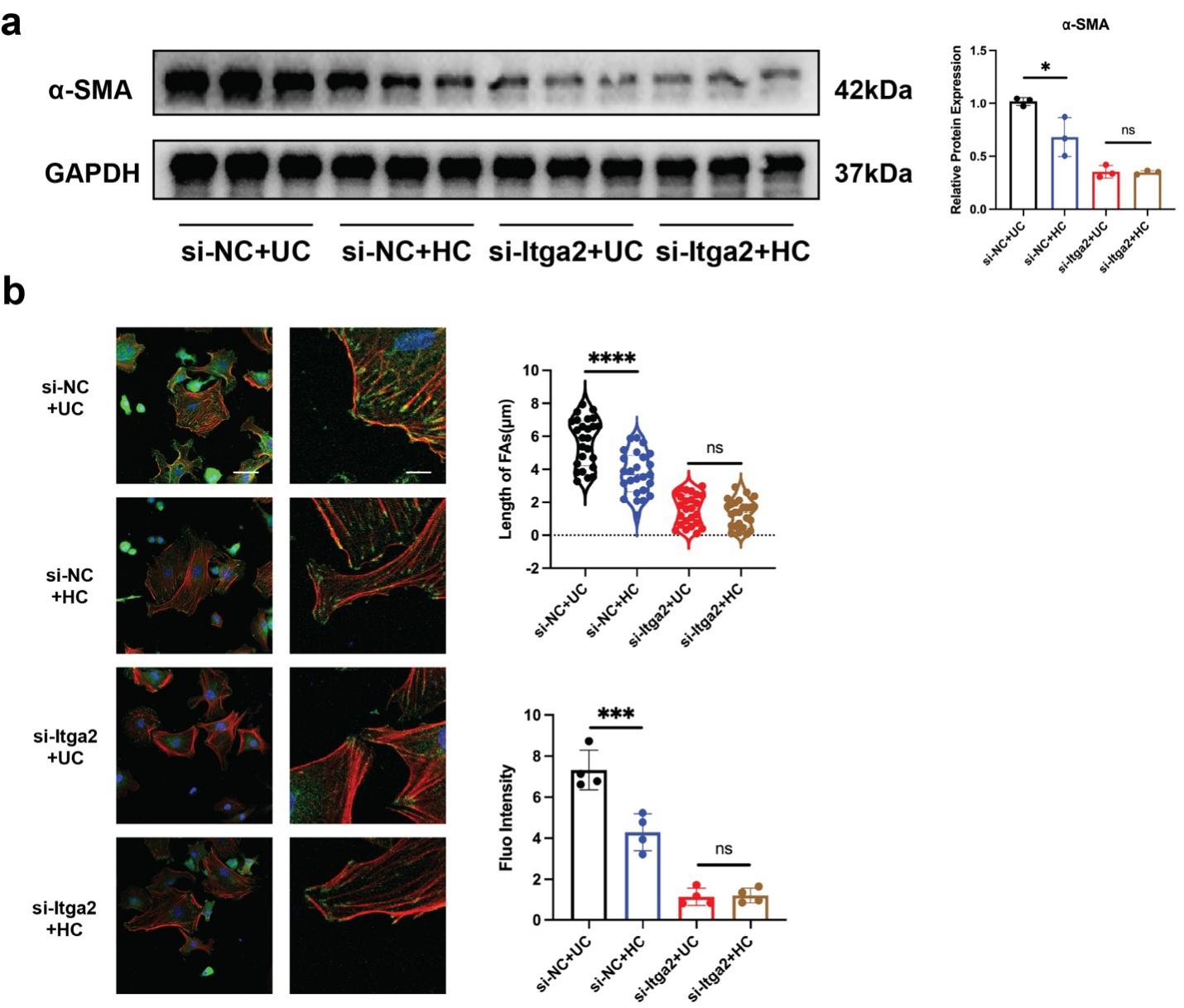


**Figure. S15 Knockdown of Itga2 resulted in similar FMT and FAs reduction effects to Itgb1 knockdown.**

**a** Western blot analysis of α-SMA after Itga2 knockdown between HC and UC (n = 3). **b** Representative immunofluorescence images and quantification of FAs length (n ≥ 20) and fluorescence intensity (n = 4) in Itga2 knockdown NMCFs on UC, HC. Scale bar, left 50µm, right 10µm. For all experiments, error bars represent the mean ± SD. *P < 0.05, **P < 0.01, ***P < 0.001, ****P < 0.0001.


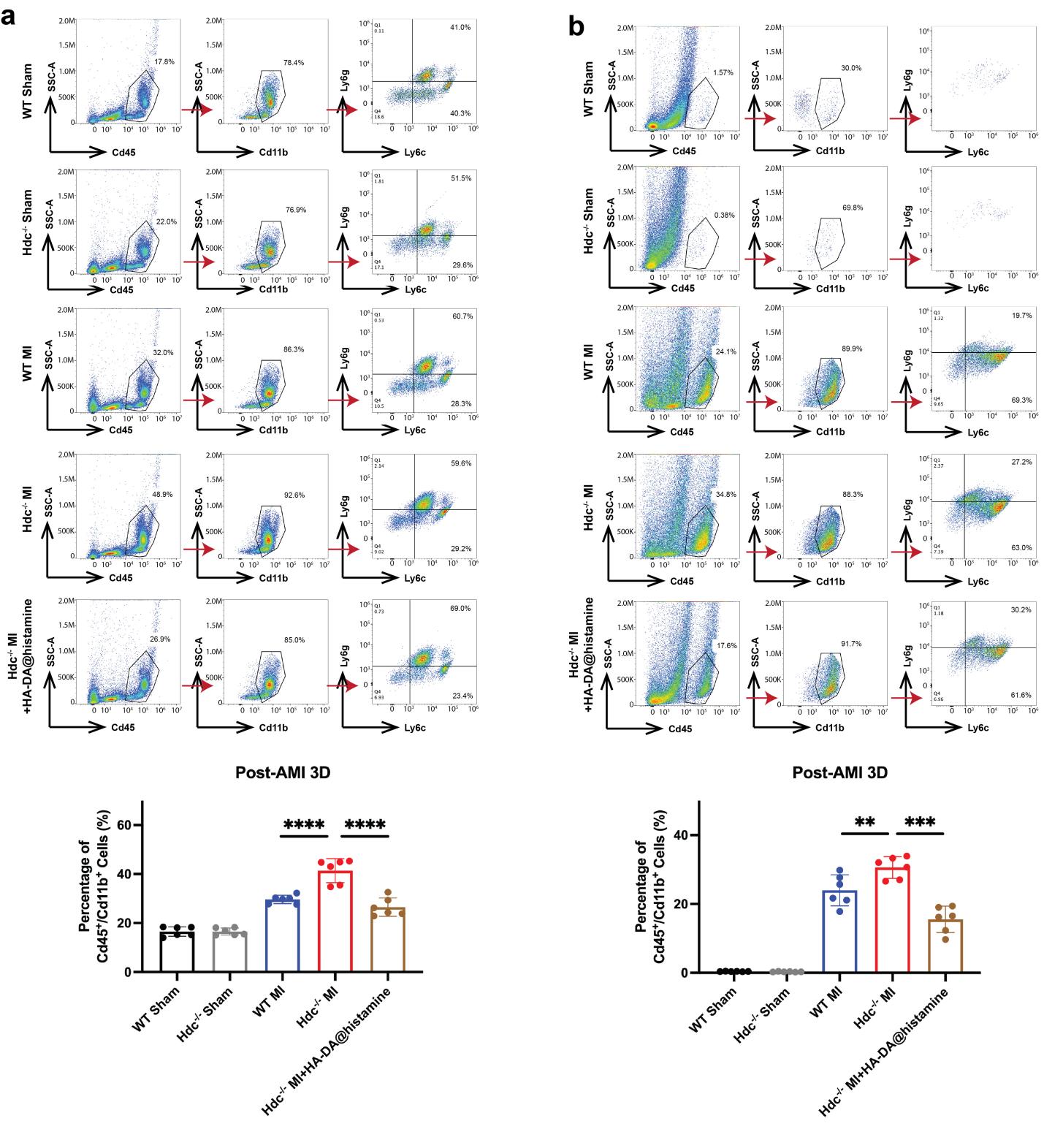


**Figure S16. Systemic immune cell recruitment and local immune cell infiltration were markedly reduced after delivery of HA-DA@histamine.**

**a** Representative image and statistical analysis of flow cytometry of peripheral blood immune cell (n = 6). **b** Representative image and statistical analysis of flow cytometry of myocardial tissue distal to the ligation site (n = 6). For all experiments, error bars represent the mean ± SD. *P < 0.05, **P < 0.01, ***P < 0.001, ****P < 0.0001.


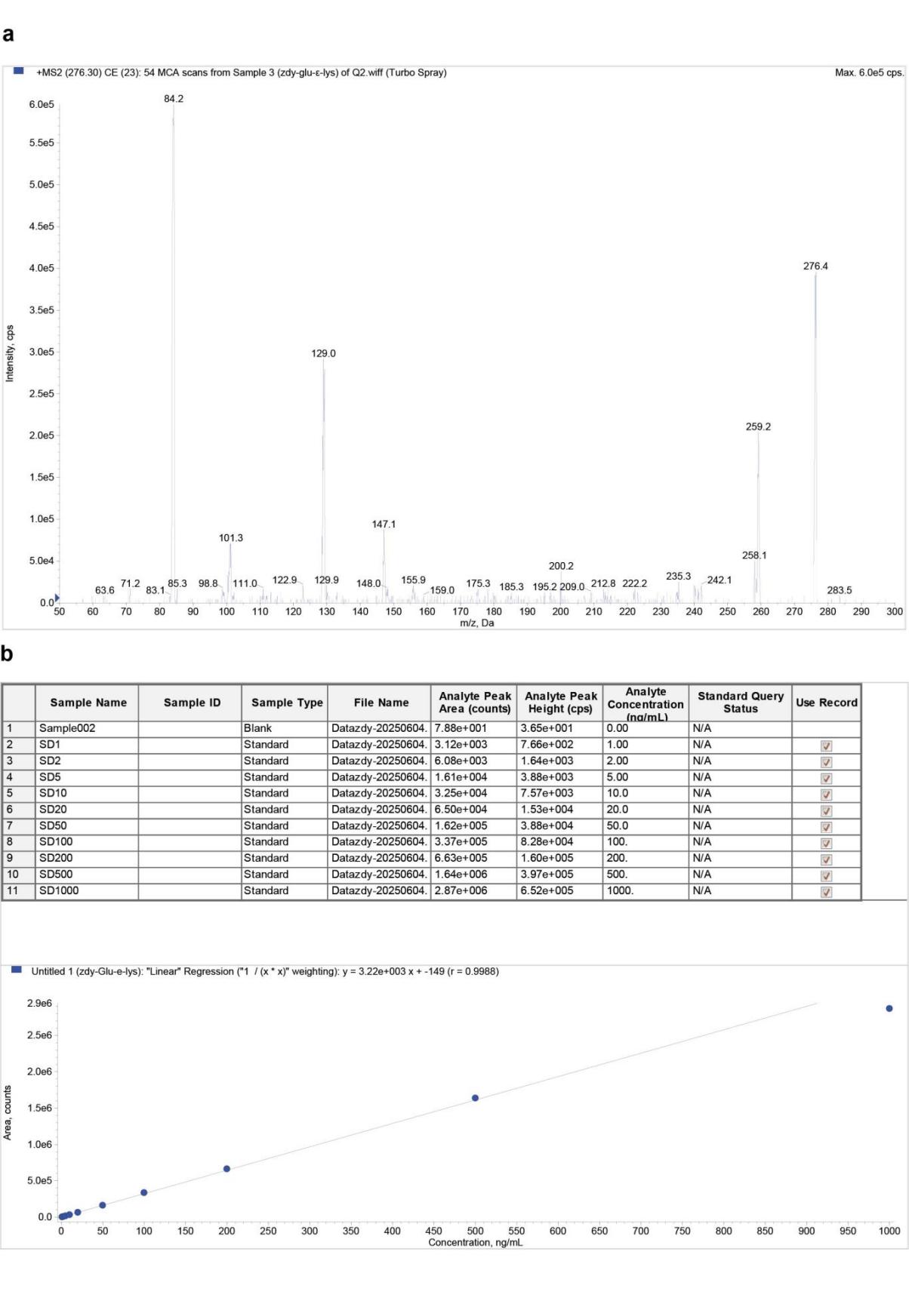


**Figure S17.** **Representative plot of AQMC assay**

**a** MS/MS spectrum of γ-Glu-ε-Lys detected by HPLC-MS/MS. **b** Standard curve for quantification of γ-Glu-ε-Lys using HPLC-MS/MS.

**
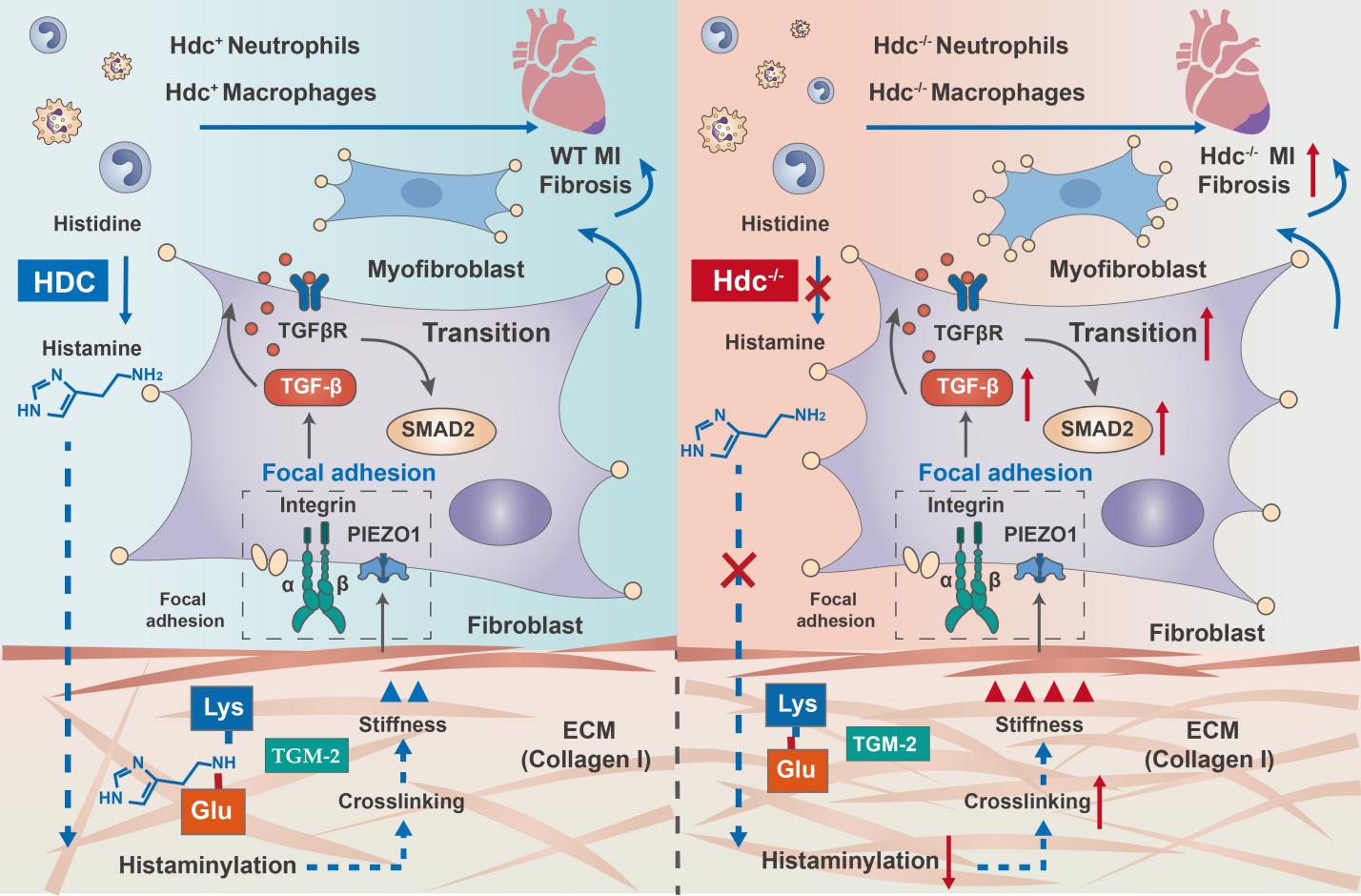
**

**Figure. S18. Schematic illustration showing the histaminylation on cardiac type I collagen,** a previously unrecognized PTM, occurs after AMI. Histaminylation competes with TGM2 for Gln residues, thereby attenuating TGM2-mediated collagen crosslinking and altering matrix mechanical properties. These changes downregulate the Piezo1/Itgb1 signaling pathway and suppress FMT, ultimately contributing to the attenuation of post-AMI cardiac fibrosis. This figure was generated using Adobe Illustrator.

**Table S1. Peptides sequences of histaminylatied Glu residues and positions pooled into analysis**

| **Sequences** | **Position** |
| --- | --- |
| PPGLGGNFASQ#MSYGYDEKSA | 152 |
| GSPGENGAPGQ#MGPRGLPGER | 289 |
| FPGARGPSGPQ#GPSGPPGPKG | 410 |
| PAGKDGEAGAQ#GAPGPAGPAG | 608 |
| PAGPAGERGEQ#GPAGSPGFQG | 623 |
| EQGPAGSPGFQ#GLPGPAGPPG | 632 |
| LPGPSGEPGKQ#GPSGSSGERG | 974 |
| PRGDKGETGEQ#GDRGIKGHRG | 1091 |
| IKGHRGFSGLQ#GPPGSPGSPG | 1106 |
| PPGSPGSPGEQ#GPSGASGPAG | 1118 |

# Histaminylated Gln residues

**Table S2.** **BLASTP conservation analysis of the modification sites in human collagen.**

| **Position** | **Max Score** | **Total Score** | **Query Cover** | **E value** | **Per. ident** | **Acc. Len** | **Accession** |
| --- | --- | --- | --- | --- | --- | --- | --- |
| 152 | 59.2 | 91 | 95% | 1e-11 | 90 | 1464 | NP_000079.2 |
| 289 | 67.7 | 1560 | 100% | 1e-14 | 100 | 1464 | NP_000079.2 |
| 410 | 62.6 | 1940 | 100% | 8e-13 | 95.24 | 1464 | NP_000079.2 |
| 608 | 59.6 | 1733 | 100% | 9e-12 | 95.24 | 1464 | NP_000079.2 |
| 623 | 65.5 | 1551 | 100% | 7e-14 | 100 | 1464 | NP_000079.2 |
| 632 | 66 | 1413 | 100% | 5e-14 | 100 | 1464 | NP_000079.2 |
| 974 | 61.7 | 1531 | 100% | 2e-12 | 95.24 | 1464 | NP_000079.2 |
| 1091 | 67.7 | 671 | 100% | 1e-14 | 100 | 1464 | NP_000079.2 |
| 1106 | 62.6 | 1503 | 100% | 8e-13 | 95.24 | 1464 | NP_000079.2 |
| 1118 | 60 | 1990 | 100% | 6e-12 | 95.24 | 1464 | NP_000079.2 |

Table S3. Animals (in vivo study)

| **Species** | **Background** | **Age** | **Sex** | **Source** |
| --- | --- | --- | --- | --- |
| Hdc^-/-^ mice | Balb/C | 8 weeks | male | Supplied by Professor Timothy C. Wang |
| Hdc-GFP mice | Balb/C | 8 weeks | male | Supplied by Professor Timothy C. Wang |
| Wild type mice | Balb/C | 8 weeks | male | Department of Laboratory Animal Science at Fudan University |

**Table S4. Fluorochrome-conjugated antibodies used in flow cytometry analysis**

| **Antibodies** | **Cat No.** | **Company** |
| --- | --- | --- |
| CD45 | 147709 | Biolegend |
| CD11b | 101211 | Biolegend |
| Ly6C | 128018 | Biolegend |
| Ly6G | 127616 | Biolegend |
| Cxcr2 | 149303 | Biolegend |

**Table S5. Primary antibodies used in experiments**

| **Experiments** | **Antibodies** | **Company** |
| --- | --- | --- |
| Immunofluorescence and immunohistochemistry assay | ACTA2 | Abcam |
|  | phospho-SMAD2 | Beyotime |
|  | Vinculin | Proteintech |
| Western blot | ACTA2 | Abcam |
|  | phospho-SMAD2 | Beyotime |
|  | PIEZO1 | Proteintech |
|  | ITGB1 | Proteintech |
|  | Cleaved Caspase 3 | Abclonal |
|  | GAPDH | Proteintech |
| Scar localization | Collagen I | Proteintech |

**Table S6. Primers used for qRT-PCR**

| **Species** | **Genes** | **Sequence 5’-3’** |
| --- | --- | --- |
| Mouse | Gapdh | F: AGGTCGGTGTGAACGGATTTG |
|  |  | R: GGGGTCGTTGATGGCAACA |
|  | Itgb1 | F: ATGCCAAATCTTGCGGAGAAT |
|  |  | R: TTTGCTGCGATTGGTGACATT |
|  | Piezo1 | F: TCATCATCCTTAACCACATGGTG |
|  |  | R: TGAAGACGATAGCTGTCATCCA |
|  | Acta2 | F: CCCAACTGGGACCACATGG |
|  |  | R: TACATGCGGGGGACATTGAAG |
|  | Fn1 | F: ATGTGGACCCCTCCTGATAGT |
|  |  | R: GCCCAGTGATTTCAGCAAAGG |
|  | Postn | F: CCTGCCCTTATATGCTCTGCT |
|  |  | R: AAACATGGTCAATAGGCATCACT |
|  | Col1a1 | F: GCTCCTCTTAGGGGCCACT |
|  |  | R: CCACGTCTCACCATTGGGG |
| Human | ACTA2 | F: CCGGGACTAAGACGGGAATC |
|  |  | R: TTGTCACACACCAAGGCAGT |
|  | FN1 | F: CGGTGGCTGTCAGTCAAAG |
|  |  | R: AAACCTCGGCTTCCTCCATAA |
|  | POSTN | F: CTCATAGTCGTATCAGGGGTCG |
|  |  | R: ACACAGTCGTTTTCTGTCCAC |
|  | COL1A1 | F: GAGGGCCAAGACGAAGACATC |
|  |  | R: CAGATCACGTCATCGCACAAC |
|  | GAPDH | F: GAAGGTGAAGGTCGGAGTC |
|  |  | R: AAGATGGTGATGGGATTTC |

**Table S7. Other reagents used in the study**

| **Experiments** | **Reagent** | **Company** |
| --- | --- | --- |
| Collagen extraction | Pepsin | Sigma Aldrich |
|  | Acetic acid | Macklin |
| Collagen matrix construction | Recombinant TGM2 | Zedira |
|  | CaCl_2_ | Macklin |
|  | Tris-base | Servicebio |
|  | Rat tail collagen | Corning |
| Fluorescent staining | Phalloidin | Beyotime |
|  | DAPI | Beyotime |
| Secondary Antibodies | HRP-conjugated Goat Anti-Rabbit IgG(H+L) | Proteintech |
|  | HRP-conjugated Goat Anti-Mouse IgG(H+L) | Proteintech |
|  | Alexa Fluor® 594-conjugated Goat Anti-Rabbit IgG (H+L) | Servicebio |
|  | Alexa Fluor® 488-conjugated Goat Anti-Rabbit IgG (H+L) | Servicebio |
|  | Alexa Fluor® 594-conjugated Goat Anti-Mouse IgG (H+L) | Servicebio |
|  | Alexa Fluor® 488-conjugated Goat Anti-Mouse IgG (H+L) | Servicebio |
| Piezo1 related treatments | Yoda1 | MCE |
|  | GsMTx4 | MCE |

Movie S1.

A representative video of an integrated fluorescence microscopy with a uniaxial cell stretcher

Data S1. (separate file)

LC-MS/MS results of type I collagen α1 chain extracted from infarcted mouse heart

Data S2. (separate file)

LC-MS/MS results of type I collagen α2 chain extracted from infarcted mouse heart

Data S3. (separate file)

LC-MS/MS results of type I collagen α1 chain from in vitro reconstructed rat tail type I collagen α1 chain

Data S4. (separate file)

LC-MS/MS results of type I collagen α1 chain from in vitro reconstructed rat tail type I collagen α2 chain

**Data S5. (separate file)**

Directly detection of histaminylation in rat-tail type I collagen

1 Gao, E. et al. A novel and efficient model of coronary artery ligation and myocardial infarction in the mouse. Circ Res 107, 1445-1453 (2010). https://doi.org/10.1161/CIRCRESAHA.110.223925

1 Gao, E. et al. A novel and efficient model of coronary artery ligation and myocardial infarction in the mouse. Circ Res 107, 1445-1453 (2010). https://doi.org/10.1161/CIRCRESAHA.110.223925

2 Feng, J. et al. Versican Promotes Cardiomyocyte Proliferation and Cardiac Repair. Circulation 149, 1004-1015 (2024). https://doi.org/10.1161/CIRCULATIONAHA.123.066298

.

1 Gao, E. et al. A novel and efficient model of coronary artery ligation and myocardial infarction in the mouse. Circ Res 107, 1445-1453 (2010). https://doi.org/10.1161/CIRCRESAHA.110.223925

2 Feng, J. et al. Versican Promotes Cardiomyocyte Proliferation and Cardiac Repair. Circulation 149, 1004-1015 (2024). https://doi.org/10.1161/CIRCULATIONAHA.123.066298

3 Lyu, C. et al. Advanced glycation end-products as mediators of the aberrant crosslinking of extracellular matrix in scarred liver tissue. Nat Biomed Eng 7, 1437-1454 (2023). https://doi.org/10.1038/s41551-023-01019-z

1 Gao, E. et al. A novel and efficient model of coronary artery ligation and myocardial infarction in the mouse. Circ Res 107, 1445-1453 (2010). https://doi.org/10.1161/CIRCRESAHA.110.223925

2 Feng, J. et al. Versican Promotes Cardiomyocyte Proliferation and Cardiac Repair. Circulation 149, 1004-1015 (2024). https://doi.org/10.1161/CIRCULATIONAHA.123.066298

3 Lyu, C. et al. Advanced glycation end-products as mediators of the aberrant crosslinking of extracellular matrix in scarred liver tissue. Nat Biomed Eng 7, 1437-1454 (2023). https://doi.org/10.1038/s41551-023-01019-z

4 Cortes-Medina, M. et al. Chondroitin sulfate, dermatan sulfate, and hyaluronic acid differentially modify the biophysical properties of collagen-based hydrogels. Acta Biomater 174, 116-126 (2024). https://doi.org/10.1016/j.actbio.2023.12.018

1 Gao, E. et al. A novel and efficient model of coronary artery ligation and myocardial infarction in the mouse. Circ Res 107, 1445-1453 (2010). https://doi.org/10.1161/CIRCRESAHA.110.223925

2 Feng, J. et al. Versican Promotes Cardiomyocyte Proliferation and Cardiac Repair. Circulation 149, 1004-1015 (2024). https://doi.org/10.1161/CIRCULATIONAHA.123.066298

3 Lyu, C. et al. Advanced glycation end-products as mediators of the aberrant crosslinking of extracellular matrix in scarred liver tissue. Nat Biomed Eng 7, 1437-1454 (2023). https://doi.org/10.1038/s41551-023-01019-z

4 Cortes-Medina, M. et al. Chondroitin sulfate, dermatan sulfate, and hyaluronic acid differentially modify the biophysical properties of collagen-based hydrogels. Acta Biomater 174, 116-126 (2024). https://doi.org/10.1016/j.actbio.2023.12.018

5 Hao, Y. et al. Integrated analysis of multimodal single-cell data. Cell 184, 3573-3587 e3529 (2021). https://doi.org/10.1016/j.cell.2021.04.048

1 Gao, E. et al. A novel and efficient model of coronary artery ligation and myocardial infarction in the mouse. Circ Res 107, 1445-1453 (2010). https://doi.org/10.1161/CIRCRESAHA.110.223925

2 Feng, J. et al. Versican Promotes Cardiomyocyte Proliferation and Cardiac Repair. Circulation 149, 1004-1015 (2024). https://doi.org/10.1161/CIRCULATIONAHA.123.066298

3 Lyu, C. et al. Advanced glycation end-products as mediators of the aberrant crosslinking of extracellular matrix in scarred liver tissue. Nat Biomed Eng 7, 1437-1454 (2023). https://doi.org/10.1038/s41551-023-01019-z

4 Cortes-Medina, M. et al. Chondroitin sulfate, dermatan sulfate, and hyaluronic acid differentially modify the biophysical properties of collagen-based hydrogels. Acta Biomater 174, 116-126 (2024). https://doi.org/10.1016/j.actbio.2023.12.018

5 Hao, Y. et al. Integrated analysis of multimodal single-cell data. Cell 184, 3573-3587 e3529 (2021). https://doi.org/10.1016/j.cell.2021.04.048

6 Bailey, T. L., Johnson, J., Grant, C. E. & Noble, W. S. The MEME Suite. Nucleic Acids Res 43, W39-49 (2015). https://doi.org/10.1093/nar/gkv416

1 Gao, E. et al. A novel and efficient model of coronary artery ligation and myocardial infarction in the mouse. Circ Res 107, 1445-1453 (2010). https://doi.org/10.1161/CIRCRESAHA.110.223925

2 Feng, J. et al. Versican Promotes Cardiomyocyte Proliferation and Cardiac Repair. Circulation 149, 1004-1015 (2024). https://doi.org/10.1161/CIRCULATIONAHA.123.066298

3 Lyu, C. et al. Advanced glycation end-products as mediators of the aberrant crosslinking of extracellular matrix in scarred liver tissue. Nat Biomed Eng 7, 1437-1454 (2023). https://doi.org/10.1038/s41551-023-01019-z

4 Cortes-Medina, M. et al. Chondroitin sulfate, dermatan sulfate, and hyaluronic acid differentially modify the biophysical properties of collagen-based hydrogels. Acta Biomater 174, 116-126 (2024). https://doi.org/10.1016/j.actbio.2023.12.018

5 Hao, Y. et al. Integrated analysis of multimodal single-cell data. Cell 184, 3573-3587 e3529 (2021). https://doi.org/10.1016/j.cell.2021.04.048

6 Bailey, T. L., Johnson, J., Grant, C. E. & Noble, W. S. The MEME Suite. Nucleic Acids Res 43, W39-49 (2015). https://doi.org/10.1093/nar/gkv416

7 Crooks, G. E., Hon, G., Chandonia, J. M. & Brenner, S. E. WebLogo: a sequence logo generator. Genome Res 14, 1188-1190 (2004). https://doi.org/10.1101/gr.849004

1 Gao, E. et al. A novel and efficient model of coronary artery ligation and myocardial infarction in the mouse. Circ Res 107, 1445-1453 (2010). https://doi.org/10.1161/CIRCRESAHA.110.223925

2 Feng, J. et al. Versican Promotes Cardiomyocyte Proliferation and Cardiac Repair. Circulation 149, 1004-1015 (2024). https://doi.org/10.1161/CIRCULATIONAHA.123.066298

3 Lyu, C. et al. Advanced glycation end-products as mediators of the aberrant crosslinking of extracellular matrix in scarred liver tissue. Nat Biomed Eng 7, 1437-1454 (2023). https://doi.org/10.1038/s41551-023-01019-z

4 Cortes-Medina, M. et al. Chondroitin sulfate, dermatan sulfate, and hyaluronic acid differentially modify the biophysical properties of collagen-based hydrogels. Acta Biomater 174, 116-126 (2024). https://doi.org/10.1016/j.actbio.2023.12.018

5 Hao, Y. et al. Integrated analysis of multimodal single-cell data. Cell 184, 3573-3587 e3529 (2021). https://doi.org/10.1016/j.cell.2021.04.048

6 Bailey, T. L., Johnson, J., Grant, C. E. & Noble, W. S. The MEME Suite. Nucleic Acids Res 43, W39-49 (2015). https://doi.org/10.1093/nar/gkv416

7 Crooks, G. E., Hon, G., Chandonia, J. M. & Brenner, S. E. WebLogo: a sequence logo generator. Genome Res 14, 1188-1190 (2004). https://doi.org/10.1101/gr.849004

8 Liu, L. et al. Matrix-transmitted paratensile signaling enables myofibroblast-fibroblast cross talk in fibrosis expansion. Proc Natl Acad Sci U S A 117, 10832-10838 (2020). https://doi.org/10.1073/pnas.1910650117

1 Gao, E. et al. A novel and efficient model of coronary artery ligation and myocardial infarction in the mouse. Circ Res 107, 1445-1453 (2010). https://doi.org/10.1161/CIRCRESAHA.110.223925

2 Feng, J. et al. Versican Promotes Cardiomyocyte Proliferation and Cardiac Repair. Circulation 149, 1004-1015 (2024). https://doi.org/10.1161/CIRCULATIONAHA.123.066298

3 Lyu, C. et al. Advanced glycation end-products as mediators of the aberrant crosslinking of extracellular matrix in scarred liver tissue. Nat Biomed Eng 7, 1437-1454 (2023). https://doi.org/10.1038/s41551-023-01019-z

4 Cortes-Medina, M. et al. Chondroitin sulfate, dermatan sulfate, and hyaluronic acid differentially modify the biophysical properties of collagen-based hydrogels. Acta Biomater 174, 116-126 (2024). https://doi.org/10.1016/j.actbio.2023.12.018

5 Hao, Y. et al. Integrated analysis of multimodal single-cell data. Cell 184, 3573-3587 e3529 (2021). https://doi.org/10.1016/j.cell.2021.04.048

6 Bailey, T. L., Johnson, J., Grant, C. E. & Noble, W. S. The MEME Suite. Nucleic Acids Res 43, W39-49 (2015). https://doi.org/10.1093/nar/gkv416

7 Crooks, G. E., Hon, G., Chandonia, J. M. & Brenner, S. E. WebLogo: a sequence logo generator. Genome Res 14, 1188-1190 (2004). https://doi.org/10.1101/gr.849004

8 Liu, L. et al. Matrix-transmitted paratensile signaling enables myofibroblast-fibroblast cross talk in fibrosis expansion. Proc Natl Acad Sci U S A 117, 10832-10838 (2020). https://doi.org/10.1073/pnas.1910650117

9 Wang, Y. et al. Versatile dopamine-functionalized hyaluronic acid-recombinant human collagen hydrogel promoting diabetic wound healing via inflammation control and vascularization tissue regeneration. Bioact Mater 35, 330-345 (2024). https://doi.org/10.1016/j.bioactmat.2024.02.010

**Data S6. (separate file)**

Original and uncropped films of Western blots
