# Supplementary figures and images for: Histaminylation alters collagen matrix mechanics and attenuates cardiac fibrosis post-myocardial infarction via mechanotransduction signaling axis
Source: Signal Transduct Target Ther. 2026 Jun 11;11:229. doi: 10.1038/s41392-026-02721-5 (PMC13254370; doi:10.1038/s41392-026-02721-5)

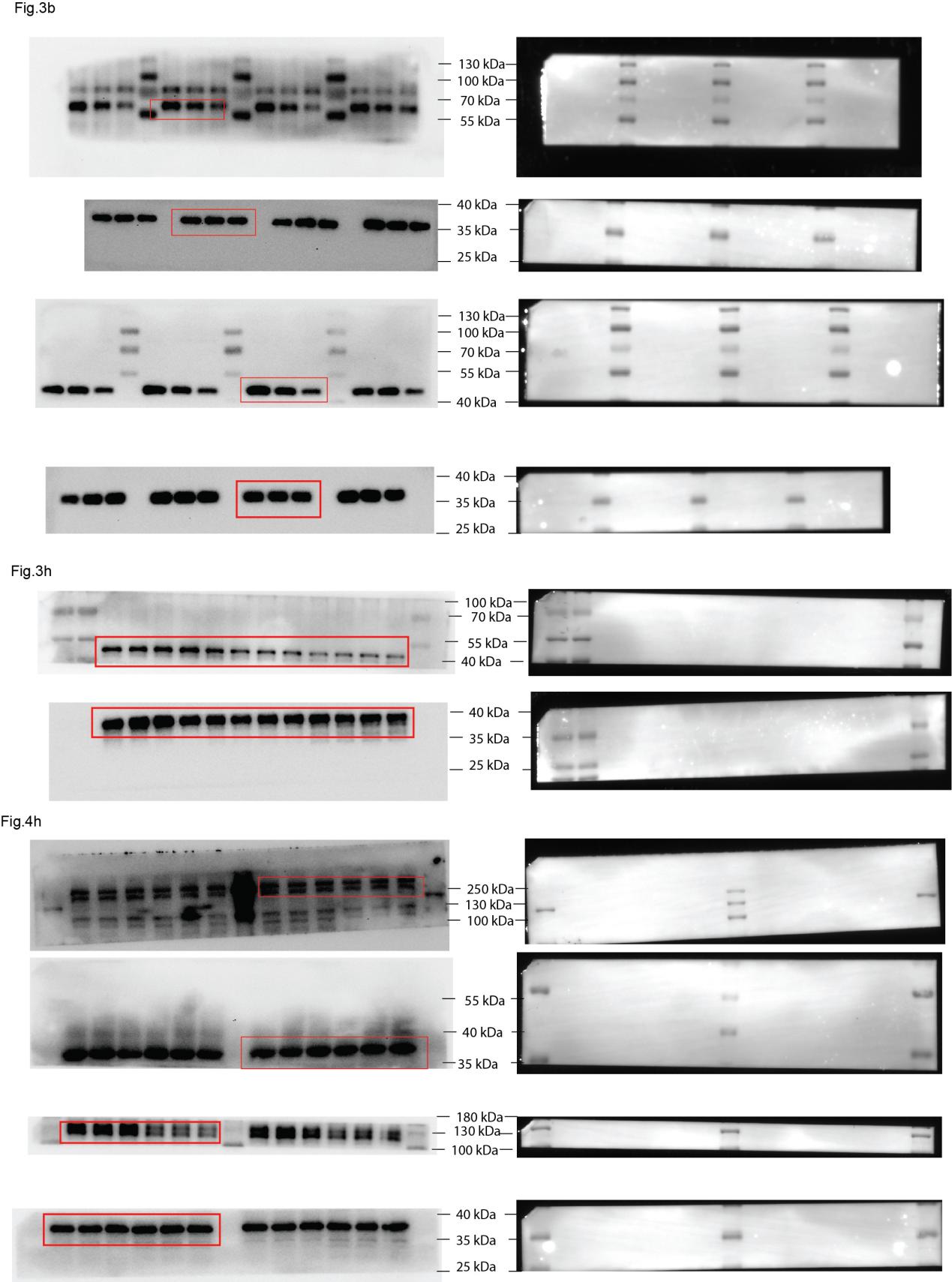

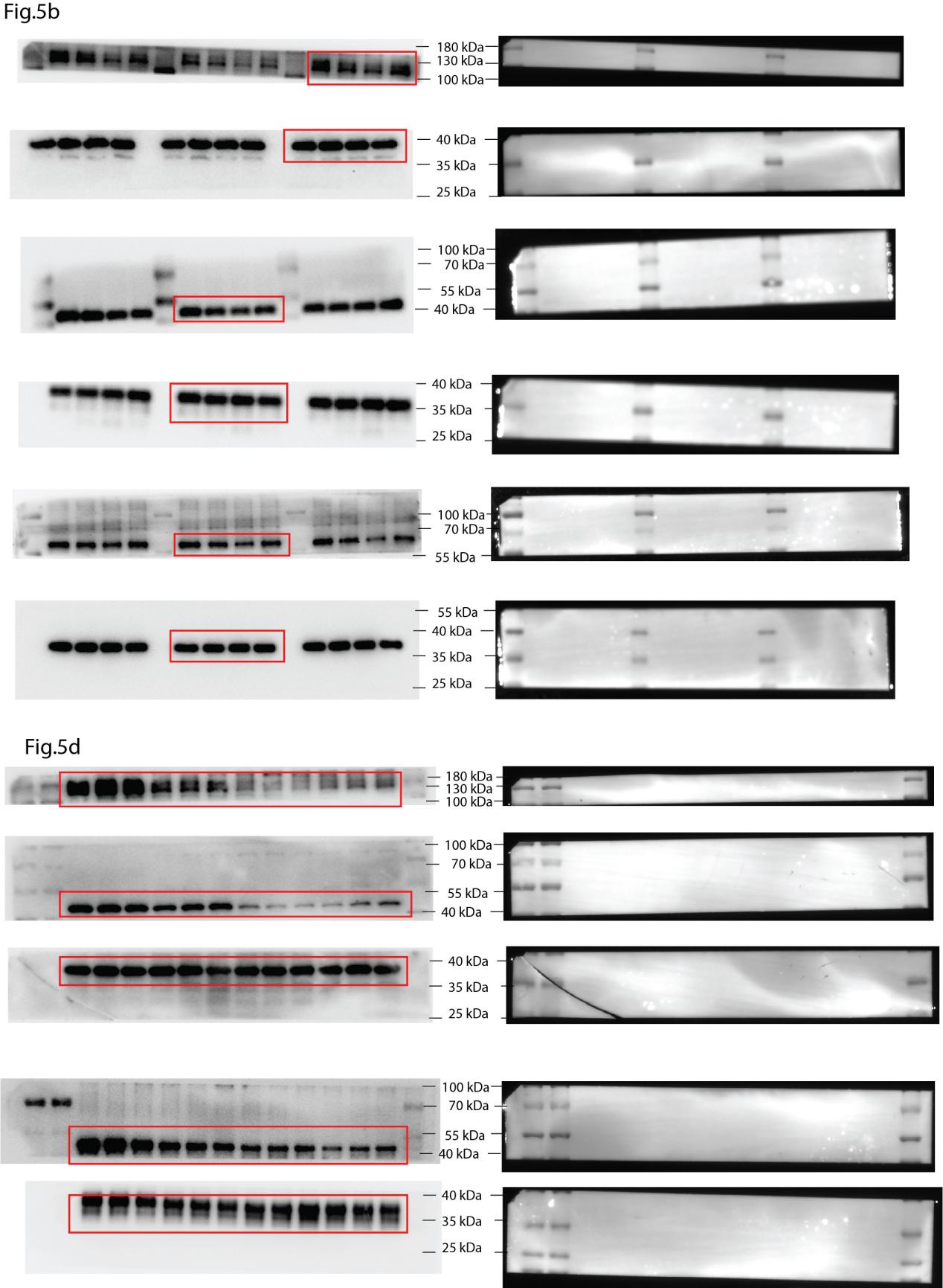

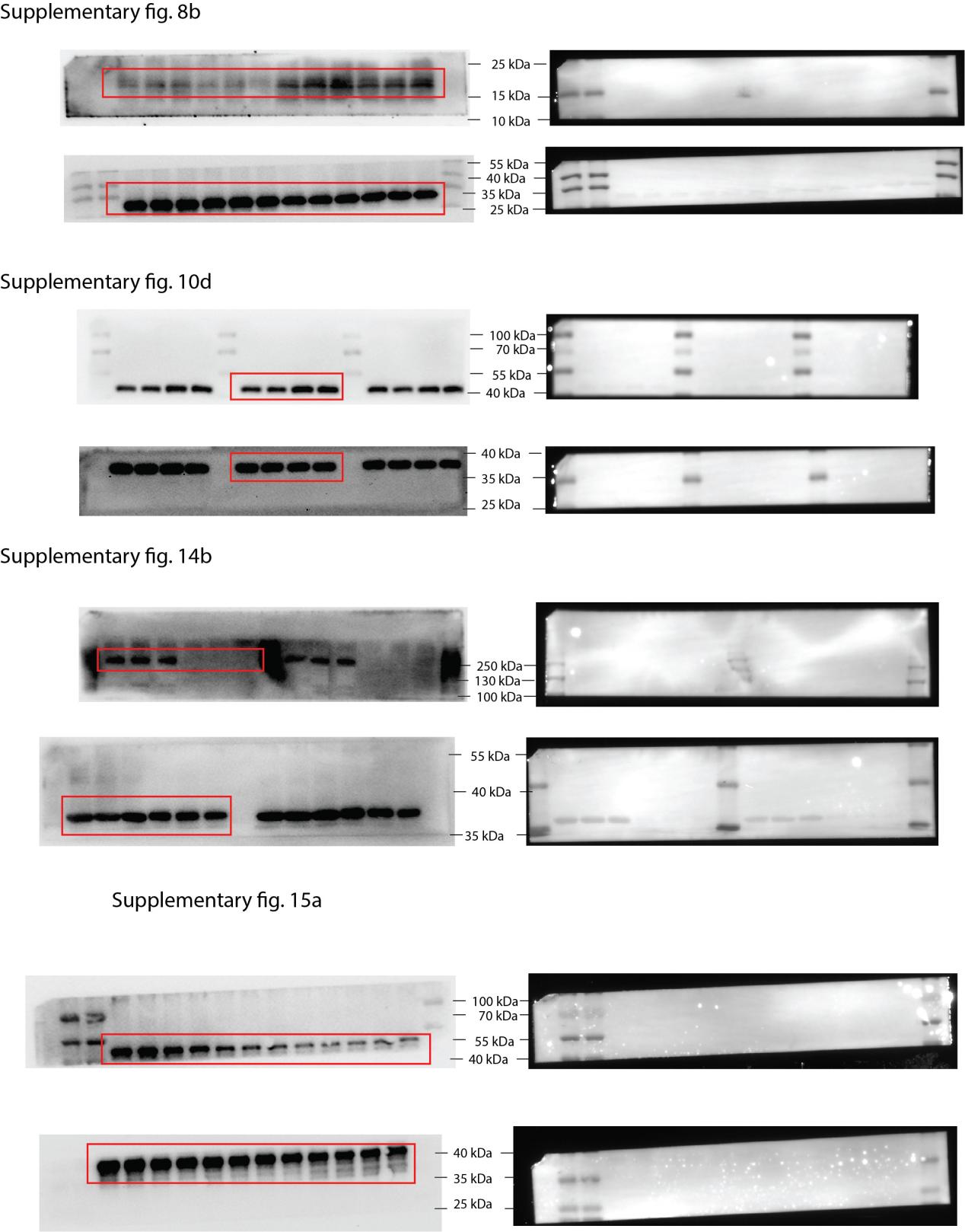

Supplement: Supplementary file 8 — Data S6 [file 41392_2026_2721_MOESM8_ESM.docx]
